# The epidemiological landscape of anemia in women of reproductive age in sub-Saharan Africa

**DOI:** 10.1038/s41598-021-91198-z

**Published:** 2021-06-07

**Authors:** Esteban Correa-Agudelo, Hae-Young Kim, Godfrey N. Musuka, Zindoga Mukandavire, F. DeWolfe Miller, Frank Tanser, Diego F. Cuadros

**Affiliations:** 1 Health Geography and Disease Modeling Laboratory, University of Cincinnati, Cincinnati, OH 45221 USA; 2 Department of Geography and Geographic Information Science, University of Cincinnati, Cincinnati, USA; 3 Africa Health Research Institute, Durban, KwaZulu-Natal South Africa; 4 KwaZulu-Natal Research Innovation and Sequencing Platform (KRISP), Durban, KwaZulu-Natal South Africa; 5 Department of Population Health, New York University Grossman School of Medicine, New York, USA; 6 ICAP at Columbia University, Harare, Zimbabwe; 7 Centre for Data Science, Coventry University, Coventry, UK; 8 School of Computing, Electronics and Mathematics, Coventry University, Coventry, UK; 9 Department of Tropical Medicine and Medical Microbiology and Pharmacology, University of Hawaii, Honolulu, HI USA; 10 Lincoln International Institute for Rural Health, University of Lincoln, Lincoln, UK; 11 School of Nursing and Public Health, College of Health Sciences, University of KwaZulu-Natal, Durban, South Africa; 12 Department of Population Health, London School of Hygiene and Tropical Medicine, London, UK

**Keywords:** Diseases, Public health, Epidemiology, Risk factors

## Abstract

The role of geographical disparities of health-related risk factors with anemia are poorly documented for women of reproductive age in sub-Saharan Africa (SSA). We aimed to determine the contribution of potential factors and to identify areas at higher risk of anemia for women in reproductive age in SSA. Our study population comprised 27 nationally representative samples of women of reproductive age (15–49) who were enrolled in the Demographic and Health Surveys and conducted between 2010 and 2019 in SSA. Overall, we found a positive association between being anemic and the ecological exposure to malaria incidence [adjusted odds ratio (AOR) = 1.02, 95% confidence interval (CI) 1.02–1.02], and HIV prevalence (AOR = 1.01, CI 1.01–1.02). Women currently pregnant or under deworming medication for the last birth had 31% (AOR = 1.31, CI 1.24–1.39) and 5% (AOR = 1.05, CI 1.01–1.10) higher odds of having anemia, respectively. Similarly, women age 25–34 years old with low education, low income and living in urban settings had higher odds of having anemia. In addition, underweight women had 23% higher odds of suffering anemia (AOR = 1.23, CI 1.15–1.31). Females with low levels of education and wealth index were consistently associated with anemia across SSA. Spatial distribution shows increased risk of anemia in Central and Western Africa. Knowledge about the contribution of known major drivers and the spatial distribution of anemia risk can mitigate operational constraints and help to design geographically targeted intervention programs in SSA.

## Introduction

Anemia is a major worldwide blood disorder related to an abnormally low concentration of hemoglobin protein in red blood cells (RBC). There are several conditions that alter RBC morphology and hemoglobin concentration including iron-deficiency, genetic mutations (e.g. sickle cell), abrupt blood loss, or infectious diseases^1^. According to the Global Burden of Disease (GBD), anemia affects 27% of the world’s population (1.93 billion), causing 8.8% of the total disability for all conditions in 2010. Sub-Saharan Africa (SSA) is a region especially vulnerable to anemia, with an estimated 190 million cases occurring in SSA countries^2^. Preschool children are at the highest risk, contributing to 43% of total anemia cases, followed by women of reproductive age (29% anemic in non-pregnant and 38% in pregnant women). Without proper treatment, anemia can cause numerous health outcomes in adults including fatigue, low work productivity, and heart failure. In children, anemia can affect cognitive and motor development, and directly contribute to child and maternal mortality^3^. In SSA, anemia frequently co-occur with other health conditions and comorbidities, including infectious diseases such as malaria, schistosomiasis, and HIV^4–7^.

Previous studies have shown geographic and demographic disparity of anemia^2,8^. Some studies have assessed the geographical variation of anemia and the contribution of malnutrition, malaria, and helminth infections in preschool children in SSA^9^, and among male populations in India^10^. Another study reported geographical association of anemia with malaria deaths in SSA^11^. Although these studies showed evidence of the spatial variation of anemia, they were geographically and epidemiologically limited, with scarce information of vulnerable populations like women, or related comorbidities in SSA. In terms of control strategies, large efforts in Africa have been focused on nutrition-related interventions including iron supplementation and food fortification programs^12^. Other strategies from the United Nations Children's Fund (UNICEF) and World Health Organization (WHO) have focused on controlling infectious diseases through deworming, malaria prevention (insecticide-treated net [ITN]) and water and sanitation agenda^13,14^. However, the high burden of anemia in SSA and the presence other health-related conditions require additional efforts from governments to identify, track, and tailor interventions on vulnerable populations and geographic areas where the burden of anemia is concentrated. Therefore, this study aims to: (a) assess the contribution of potential risks factors and comorbidities associated with anemia for women in reproductive age; and (b) identify the locations where the burden of the disease is concentrated in SSA. We hypothesized that women in reproductive age living in areas with high prevalence of comorbidities and poor sanitation conditions are adversely affected by anemia in SSA.

## Methods

### Study area and data sources

Our study used observational data from the population-based Demographic Health Survey (DHS) conducted between 2010 and 2019 in 27 countries in SSA. Data for anemia status and associated socioeconomic and demographic factors for 384,291 women of reproductive age (15–49 years) were obtained from the most recent standard and continuous DHS, including: Burundi (BDI), Benin (BEN), Burkina Faso (BFA), Ivory Coast (CIV), Cameroon (CMR), Democratic Republic of Congo (COD), Congo (COG), Ethiopia (ETH), Gabon (GAB), Ghana (GHA), Guinea (GIN), The Gambia (GMB), Lesotho (LSO), Mali (MLI), Mozambique (MOZ), Malawi (MWI), Namibia (NAM), Niger (NER), Nigeria (NGA), Rwanda (RWA), Senegal (SEN), Sierra Leone (SLE), Togo (TGO), Tanzania (TZA), Uganda (UGA), South Africa (ZAF) and Zimbabwe (ZWE). Data on two comorbidities, malaria and HIV, were obtained from the Malaria Atlas Project (MAP) and the Institute for Health Metrics and Evaluations (IHME)^15,16^. Inequality in accessibility to health resources was quantified using predicted travel time in hours to the nearest city from MAP^17^. The unit of analysis (Admin1 territory) was derived from the spatial data repository for DHS (http://spatialdata.dhsprogram.com/) to match each country’s sampling design and site locations.

### Study variables

The primary outcome of interest was the anemia status. Women were grouped into two categories: “*non-anemic”*, and *“anemic”*. DHS determines anemia status by measuring hemoglobin concentration in blood samples adjusted by altitude in grams per deciliter (g/dl) as defined in the WHO’s guideline^18^. For pregnant women, hemoglobin levels below 10.9 g/dl were considered anemic. For non-pregnant women, hemoglobin levels below 11.9 g/dl were considered anemic.

Due to the strong co-occurrence of anemia with other health conditions in SSA, we extracted and averaged the 2015 malaria incidence (*Plasmodium falciparum* incidence rate [*Pf*IR]), and 2015 HIV prevalence estimates at the minimum unit of analysis available in DHS (Admin1). For maternity history, we included: pregnancy status, iron supplementation, and deworming during pregnancy^19,20^. Sociodemographic covariates included age, type of residence, education, source of drinking water, wealth index, body mass index (BMI), and smoking cigarettes. Covariates were classified using appropriate levels: pregnancy status (yes or no/unsure), iron supplementation during pregnancy (yes or no), and deworming during pregnancy (yes or no), age groups (15–24, 25–34, 35–44, 45 +), type of residence (urban or rural), education (no education, primary/secondary or higher), source of drinking water (improved or unimproved), wealth index combined (poorest, poorer, middle, richer or richest), BMI (underweight or normal/obese), and smoking cigarettes (yes or no). Inequality in accessibility to health resources was assessed using the spatial average procedure on the travel time surface for each Admin1. This study follows the guidelines of the Strengthening the Reporting of Observational Studies in Epidemiology (STROBE)^21^. Additional information about each country’s DHS studies and variables, and STROBE checklist is included in Supplemental Material (See Appendix A and B).

### Statistical analysis

Population surveys from each country were combined into a full dataset, preserving sampling procedure. All covariates were selected according to an evidence synthesis process of relevant references^7,10,19,22–26^. To evaluate multicollinearity, a Variance Inflated Factor (VIF) was estimated including all covariates. All variables with a VIF lower than five (VIF < 5.0) were included in the final adjusted model (See Table S3). Finally, unadjusted and adjusted logistic regression models were fitted to assess the association between anemia status and the selected covariates using the full dataset. All models were weighted and adjusted accordingly to the sample design as recommended by DHS^27^. Note that only individuals who completed the survey with a valid anemia test were included in the analysis.

### Spatial risk and disease mapping

For the geographic mapping, a Bayesian spatial Poisson model was used to estimate relative risks of anemia^28^. Benefits from the model-based approach are twofold: first, they allow to introduce a reference population to obtain reliable risk estimates of a disease based on standardized incidence ratios (ratio of the observed to the expected disease counts [SIRs]). Second, disease models offer a smoothing mechanism to improve local risk estimates while avoiding extreme values of areas with small populations when incorporating mixed effects and spatial dependence (relative risk [RR]). For counts, observed anemia cases were aggregated at the minimum unit of analysis available (Admin1) following the DHS sampling procedure^27^. For the expected cases, an indirect age-standardization was included using the United Nation Adjusted Gridded Population surface for 2015 (100 m × 100 m grid resolution)^29^. This population surface was combined with the Admin1 boundaries and the 2015 World Bank estimates for population and age distribution of women aged 15–49 living in those areas to standardize the outcome across all locations^30^. Admin1 areas with a 95% Bayesian credible interval (CrI) that falls within the mean (1) indicates the same RR as expected from the reference population. Conversely, CrI > 1 or CrI < 1 were locations with low and high risk respectively. Detailed information is included in Supplemental Material (See Appendix B and C).

All maps generated in this study were created using R programming environment, version 3.6.3 (https://cran.r-project.org/bin/windows/base/old/3.6.3/) including packages *INLA*^31,32^, *SpatialEpi*^33^, *ggplot2* and *raster*.

### Ethics approval

Procedures and questionnaires for standard Demographic and Health Surveys have been reviewed and approved by the Inner-City Fund (ICF) International Institutional Review Board (IRB). The ICF International IRB ensures that the survey complies with the US Department of Health and Human Services regulations for the protection of human subjects, while the host country IRB ensures that the survey complies with laws and norms of the nation (http://dhsprogram.com/What-We-Do/Protecting-the-Privacy-of-DHS-Survey-Respondents.cfm%23sthash.Ot3N7n5m.dpuf).

### Informed consent

All DHS surveys comprised in this study included informed consent statements agreed to by the participants (https://dhsprogram.com/methodology/Protecting-the-Privacy-of-DHS-Survey-Respondents.cfm).

## Results

### Sociodemographic and socio-environmental variables

**Figure 1:**
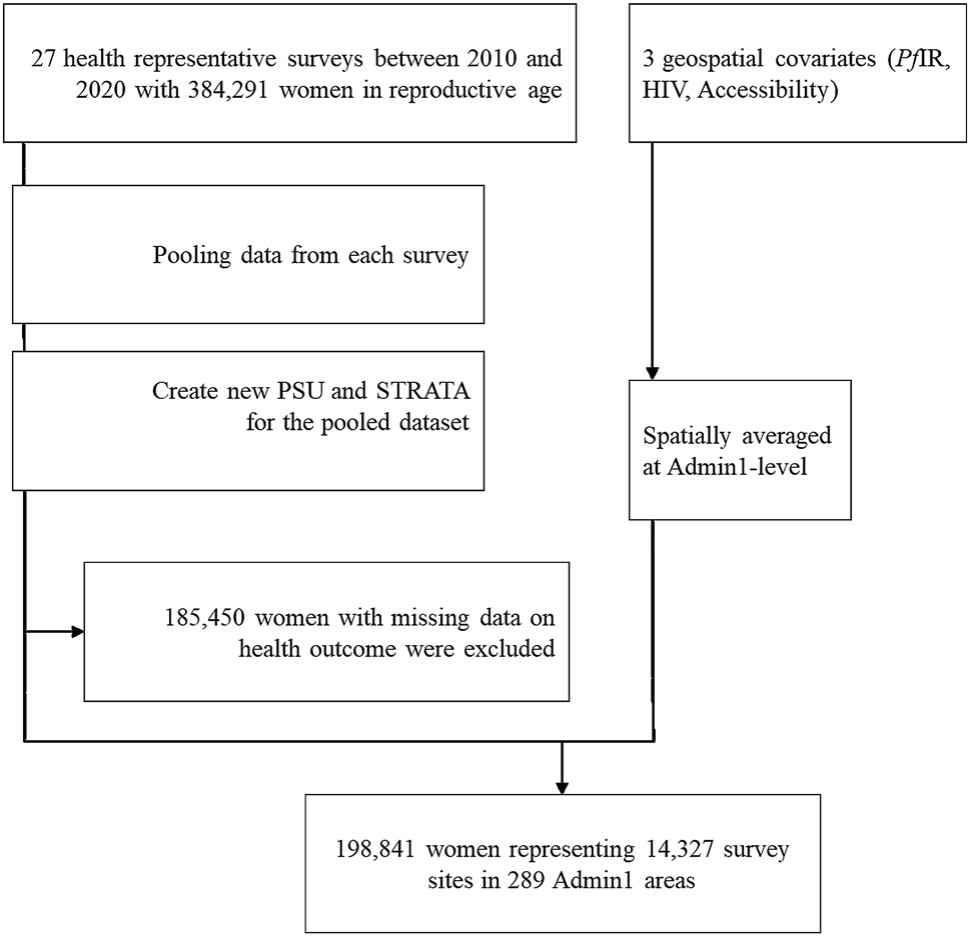
Flowchart of data identification, extraction and curation. PSU = Primary sampling unit, P*f*IR = *Plasmodium falciparum* incidence rate.

Of the 384,291 women aged 15–49, 198,841 individuals within 14,327 survey sites in 289 Admin1 areas met the inclusion criteria (had valid anemia test results and completed the survey) (Fig. 1). Table 1 presents descriptive statistics of population included in the study. The overall proportion of women with anemia was 43.3% (86,117) across the 27 countries studied. The highest prevalence was found in West (mean [µ] = 51.3, standard error [SE]:0.0), followed by Central (µ = 47.5, SE:0.01), Eastern (µ = 35.4, SE:0.0) and Southern (µ = 35.1, SE:0.01) Africa regions, respectively. Higher P*f*IR was found in anemic females compared to non-anemic females (24.3% vs 20.0%; p < 0.001). Likewise, HIV prevalence was lower in the anemic group (4.2% vs 4.7%; p < 0.01). For maternity covariates, the proportion of women in pregnancy, and the proportion of women who had received drugs for intestinal parasites were greater in the anemic group, 10.8% vs 7.8% (p < 0.001), and 23.2% vs 21.1% (p < 0.001). Low levels of education, use of unimproved source of water, and low wealth index were more common in anemic individuals, 36.9% vs 28.9% (p < 0.001), 27.3% vs 25.2% (p < 0.001), and 19.1% vs 16.5% (p < 0.001), respectively. Being underweight was more common in anemic compared to non-anemic individuals (10.7% vs 9.6%; p < 0.001). Finally, mean travel time to nearest city within Admin1 regions was lower in the anemic group (2.39 h vs 2.44 h; p = 0.027).

**Table 1:** Population characteristics by anemic status and Admin1-level covariates.

	Anemia Status				
	No anemia		Anemia		
	n	(%)	n	(%)	
Sample	112,724	(56.7)	86,117	(43.3)	
*Comorbidities*	µ	(SD)	µ	(SD)	p-value
PfIR (Admin1)	20.0	(0.09)	24.3	(0.11)	< 0.001
HIV (Admin1)	4.7	(0.03)	4.2	(0.03)	< 0.001
*Currently pregnant*					< 0.001
No or unsure	103,943	(92.2)	76,801	(89.2)	
Yes	8,808	(7.80)	9,316	(10.8)	
Age (years)	28.54	(0.03)	28.53	(0.04)	0.874
*Age-group*					
15–24	44,248	(39.3)	34,171	(39.7)	< 0.001
25–34	36,831	(32.7)	27,241	(31.6)	
35–44	23,371	(20.7)	18,438	(21.4)	
45 +	8,275	(7.3)	6267	(7.3)	
*Type of place*					
Urban	42,169	(37.4)	32,788	(38.1)	0.087
Rural	70,555	(62.6)	53,329	(61.9)	
*Education*					
No education	32,562	(28.9)	31,781	(36.9)	< 0.001
Primary	38,178	(33.9)	25,695	(29.8)	
Secundary	36,557	(32.4)	25,552	(29.7)	
Higher	5,425	(4.8)	3,084	(3.6)	
*Type of water*					
Improved source	81,609	(72.4)	60,499	(70.3)	< 0.001
Unimproved source	28,364	(25.2)	23,537	(27.3)	
Missing data	2,751	(2.4)	2,081	(2.4)	
*Wealth Index*					
Poorest	18,553	(16.5)	16,464	(19.1)	< 0.001
Poorer	20,439	(18.1)	16,695	(19.4)	
Middle	21,397	(19.0)	17,100	(19.9)	
Richer	24,031	(21.3)	17,515	(20.3)	
Richest	28,305	(25.1)	18,342	(21.3)	
*BMI*					
Obese	28,210	(25.0)	17,917	(20.8)	< 0.001
Normal	69,825	(61.9)	54,594	(63.4)	
Underweight	10,805	(9.6)	9,192	(10.7)	
Missing data	3,884	(3.4)	4,414	(5.1)	
*Smoking cigarettes*					
No	106,974	(94.9)	82,278	(95.5)	0.272
Yes	1,010	(0.9)	725	(0.8)	
Missing data	4,741	(4.2)	3,114	(3.6)	
*Iron supplementation during pregnanc*y					
No	11,626	(10.3)	8,967	(10.4)	0.363
Yes	38,168	(33.9)	28,821	(33.5)	
Missing data	62,931	(55.8)	48,329	(56.1)	
*Deworming during pregnancy*					
No	32,646	(29.0)	25,696	(29.8)	< 0.001
Yes	23,768	(21.1)	19,974	(23.2)	
Missing data	56,311	(50.0)	40,446	(47.0)	
	µ	(SD)	µ	(SD)	0.027
Accessibility to main cities (hour)	2.44	(0.02)	2.39	(0.02)	

### Multivariate analyses of risk factors for anemia

Table 2 summarizes the results from the adjusted model for the association between anemia status and the covariates in all countries. For comorbidities, the ecological exposure to a higher incidence of malaria or HIV prevalence were positively associated with having anemia. These translate to increases in the odds of having anemia for each one-unit increase in the regional P*f*IR (adjusted odds ratio [AOR] = 1.02, 95% confidence interval [CI] 1.02–1.02) and HIV prevalence (AOR = 1.01, CI 1.01–1.02), respectively. For maternity covariates, pregnancy and deworming increased the odds of having anemia by 31% (AOR = 1.31, CI 1.24–1.39) and 5% (AOR = 1.05, CI 1.01–1.10), respectively. Females between 25 and 34 years old and living in rural settings were 10% (AOR = 0.90, CI 0.86–0.94) and 20% (AOR = 0.80, CI 0.75–0.85) less likely to have anemia. Anemia more likely occurred in women with low education and low wealth index settings. Females with an unimproved source of water had 6% higher odds of having anemia (AOR = 1.06, CI 1.01–1.11). Similarly, individuals with underweight were 23% (AOR = 1.23, CI 1.15–1.31) more likely to have anemia. Smoking cigarettes and iron supplementation during pregnancy were not associated with being anemic. Lastly, women living in regions with longer distances to main cities had the decreased odds of having anemia by 1% per hour of travel time.

**Table 2:** Predictors for anemia in women of reproductive age in SSA.

	AOR	(95% CI)
*Comorbidities*		
PfIR	**1.02**	**(1.02, 1.02)**
HIV prevalence	**1.01**	**(1.01, 1.02)**
*Currently Pregnant*		
No or unsure	Ref	
Yes	**1.31**	**(1.24, 1.39)**
*Iron supplementation during pregnancy*		
No	Ref	
Yes	0.97	(0.92, 1.01)
*Deworming during pregnancy*		
No	Ref	
Yes	1.05	(1.01, 1.10)
*Age-group*		
15–24	Ref	
25–34	**0.90**	**(0.86, 0.94)**
35–44	0.97	(0.92, 1.02)
45 +	0.91	(0.81, 1.03)
*Type of place*		
Urban	Ref	
Rural	**0.80**	**(0.75, 0.85)**
*Education*		
Higher	Ref	
Primary/Secondary	**1.15**	**(1.02, 1.30)**
No education	**1.65**	**(1.46, 1.87)**
Improved source of water	Ref	
Unimproved source of water	**1.06**	**(1.01, 1.11)**
*Wealth Index*		
Richest	Ref	
Richer	**1.11**	**(1.04, 1.19)**
Middle	**1.21**	**(1.13, 1.31)**
Poorer	**1.23**	**(1.14, 1.34)**
Poorest	**1.3**4	**(1.24, 1.45)**
*BMI*		
Normal/Obese	Ref	
Underweight	**1.23**	**(1.15, 1.31)**
*Smoking cigarettes*		
No	Ref	
Yes	1.08	(0.87, 1.34)
Accessibility to main cities (hour)	**0.99**	**(0.98, 0.99)**

Notes: Boldfaced numbers indicate statistical association <0.05.

Abbreviations: AOR, adjusted odds ratio; CI, confidence interval.

### Spatial risk and disease mapping

**Figure 2:**
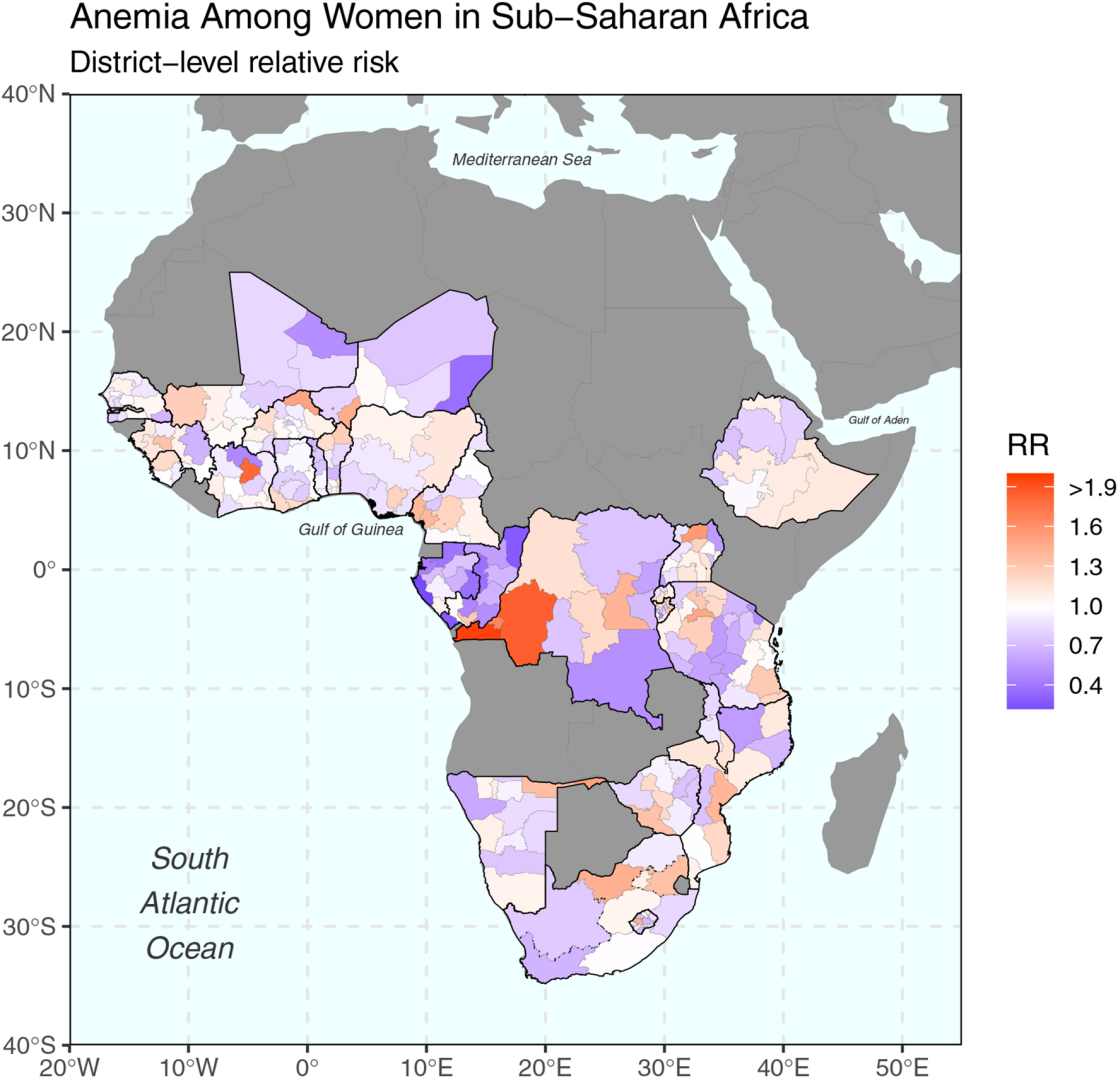
Estimated anemia risk among women in Sub-Saharan Africa (range is 0.22–7.05). Maps were generated using R programming environment, version 3.6.3 (https://cran.r-project.org/bin/windows/base/old/3.6.3/).

Figure 2 and Table S6 illustrate the RR of anemia for women in reproductive age for all countries in SSA. Overall, we found that 76 out of the 289 Admin1 locations had significantly higher risk of anemia (CrI > 1). Eastern and Western Africa accounted for 32 and 25 high-risk locations, whereas Central and Southern Africa had 14 and five, respectively. All 27 countries had at least one or more Admin1 locations which had a higher risk than average. Four countries had half or more of their regions at high-risk: COD (55%), GMB (62%), NGA (50%), and MOZ (55%). Table 3 reports the top-five Admin1 by RR. Pointe Noire (RR = 7.05, CrI 6.59, 7.53) in COG, Libreville & Port-Gentil (RR = 4.35, CrI 4.15, 4.55) in GAB, Banjul (RR = 3.93, CrI 2.89, 5.13) in GMB, Bas-Congo (RR = 1.98, CrI 1.74, 2.23) and Bandundu (RR = 1.86, CrI 1.71, 2.01) in COD. Additional details can be found in the supplemental material (See Appendix B and C).

**Table 3:** Top-five highest risk areas for anemia in SSA.

Region	Anemia Prevalence (%)	Pregnancy (%)	No formal or primary schooling (%)	Poverty (%)	Smokes cigarettes (%)	Living in rural area (%)	Accessibility to main cities (Hour)	Unimproved source of water (%)
Pointe-noire, COG	0.60	0.09	0.25	0.00	0.01	0.00	0.01	0.03
Libreville & Port-gentil, GAB	0.62	0.10	0.20	0.01	0.03	0.00	0.06	0.02
Banjul, GMB	0.52	0.07	0.37	0.00	0.00	0.00	0.22	0.01
Bas-Congo, COD	0.55	0.11	0.50	0.06	0.00	0.71	2.69	0.36
Bandundu, COD	0.38	0.13	0.49	0.22	0.00	0.79	7.96	0.69

Women in Underweight (%)	*Pf*IR (%)	HIV Prevalence (%)	Anemia RR CrI: [2.5%, 97.5%]					
0.12	0.20	3.53	7.05 [6.59, 7.53]					
0.06	0..24	3.88	4.35 [4.15, 4.55]					
0.11	0.02	1.62	3.93 [2.89, 5.13]					
0.22	0.26	0.47	1.98 [1.74, 2.23]					
0.24	0.21	0.43	1.86 [1.71, 2.01]					

## Discussion

This study provides a comprehensive characterization of health and sociodemographic determinants of anemia, and the identification of geographic areas where the burden of anemia in women of reproductive age is concentrated in SSA. We found that high prevalence of co-occurrent health conditions like malaria and HIV might exacerbate the burden of anemia in women. Women currently pregnant or under deworming medication for the last birth were more likely to have anemia. Similarly, anemia was more likely in women aged 25–34 years old, with low education, low income and living in urban settings. In addition, underweight women were 23% more likely to suffer anemia. Finally, our spatial analysis highlights high-risk areas that are worth noting for geographically targeted interventions.

Compared to the WHO latest anemia prevalence estimate in SSA (38%, CI 32.7, 45.4), our anemia estimated prevalence of 43% is slightly higher but within the WHO’s confidence intervals for Africa continent^34^. Although our regional estimates did not completely match other previous estimates^2,8^, our estimates lie within the confidence intervals of their estimates and present similar trends with very high prevalence in Central and Western Africa (> 47%). Likewise, our estimates are higher for non-pregnant (42% vs 29%) and pregnant women (51% vs 38%), compared to Stevens et al^8^. Since DHS surveys are collected in different years, each country might have different prevalences than the estimates used in WHO and other studies at the moment of the study.

Our study also highlights the co-occurrence of anemia with other highly prevalent health conditions in SSA, such as malaria and HIV. We found women living in areas with a regional P*f*IR mean of 21.4% (range 0.5–62.3) had twice the odds of having anemia. Although children aged under-five face the highest risk of malaria, low birth weight and malaria-related mortality can affect women, especially pregnant women^4^. Despite recent progress of Central and Eastern Africa in malaria prevention^35^, considerable risk persists in highly prevalent anemia areas (Central and Western Africa). Therefore, combined interventions including ITNs, antimalarial treatments, and education can foster the results not only in malaria but subsequently in anemia, especially in the high burden areas of anemia in Central and Western Africa identified in this study. Similarly, we found a significant link between HIV and anemia. Africa exhibits the highest burden of HIV (~ 25 million people living with HIV) particularly affecting specific high-risk groups (e.g., young females)^36^. In addition, the prevalence of anemia in people living with HIV might reach 80–90% under certain conditions^7^. During a lifetime, about 30% of HIV patients suffer anemia frequently due to blood loss (e.g., sarcoma), decreased or ineffective RBC production and RBC destruction. The health outcome for patients with both conditions varies from decreased survival rates to faster disease progression in HIV patients^37^. Clinical efforts including special regimens (differential diagnostic, diet, treatment and dosage) and extra monitoring for patience adherence can help to diminish anemia in HIV-positive populations.

Education attainment was associated with anemia in women, showing higher odds for females with low levels of education, which could be also linked to malnutrition and unimproved sources of drinking water. These factors could collectively be pressing manifestations of the link between poverty and anemia in SSA^8,14,38,39^. Several implications might be derived from this poverty-anemia relationship. First, low education and income levels might influence women's awareness of health issues related to unhealthy dietary habits and access to good nutritional food sources. Second, early-life underweight has later chronic disease outcomes and other disabilities in adults. In pregnancy, maternal undernutrition may result in a three-fold increased risk of low birth weight or cardiovascular diseases in adulthood^40–42^. Lastly, lack of access to an improved source of drinking water increases not only the odds of anemia but also numerous water-borne and neglected tropical diseases, including cholera, schistosomiasis, among other diseases^9^. Because poverty-related anemia possesses these critical characteristics, it would be essential for authorities to prioritize hybrid interventions based on the Sustainable Development Goals (SDGs) including scaling-up iron and fortified food interventions (2^nd^ SDG, Zero hunger), health education, drug-based deworming particularly for pregnant women and water and sanitation (6^th^ SDG, Clean water and sanitation) for greater impact on population at risk of anemia.

According to our spatial analysis, 76 subnational regions (Admin1; 26% of the total number of subnational regions included in the study) had a significantly higher risk of anemia than the SSA reference. Central and Eastern Africa each accounted for about 31% of high-risk locations. Likewise, Western and Southern Africa had about 23% and 16% of high-risk locations, respectively. At national level, all countries had at least one high-risk subnational location, and four countries had 50% or more of their regions at risk: COD (6 out of 11), GMB (5 out of 8), MOZ (6 out of 11), and NGA (3 out of 6). At Admin1-level, noteworthy is that COD (Bas-Congo, and Bandundu) and GMB (Banjul) are also present with three of the top-five highest risk locations, led by COG (Pointe Noire) and GAB (Libreville & Port-gentil). Reasons for the increased spatial risk of anemia in these territories could be manifold: first, the increased urbanization trend of Africa^43^ might explain the protective effect of rural and accessibility covariates, and why several high-risk locations are mainly urban, including the top-three regions, Pointe Noire (COG), Libreville & Port-gentil (GAB), and Banjul (GMB). Second, proportions of women in underweight exceeded overall average (9.4%) in at least four of six countries, COD (13.1%), COG (13.5%), GMB (15.1%), and NGA (11.3%) (See Table S5). Compared to the study average (8.9%), pregnancy was consistently higher in COD (12%), GAB (10.1), MOZ (11.1), and NGA (10.5). In terms of comorbidities, three of those countries accounted for nearly 40% of global malaria cases, NGA (25%), COD (12%), and MOZ (4%). In addition, COG and GAB are the only countries that exhibit low levels of household ITN ownership (< 25%) according to WHO^44^. For HIV, MOZ had one of the highest HIV prevalence (12.4%) in Eastern Africa^36^. Finally, genetic predisposition related to the sickle haemoglobin (HbS) allele in Central and Western Africa^45^ could explain COG (Pointe Noire) and GAB (Libreville & Port-gentil) risk outliers.

## Limitations

Our study had several limitations worth noting. Most of the factors included in our analysis were self-reported variables, which may lead to information bias and generate inaccuracies related to recall and possible reticence of participants in disclosing some essential information. Selection bias may be present in the sample since only selected individuals who were willing to participate in the survey and met the inclusion criteria were included in the analysis. Selection bias might have led to spurious associations and mapping errors for anemia in some countries. Another limitation is that we used the latest DHS survey conducted in each country. Therefore, some temporal differences among surveys may result in anemia estimates varying significantly over the ten years difference among all countries included. Since we restricted the study to the use of each specific DHS territory definition, geographies with subsequent subnational variation, and territories with no difference between cities and provinces might appear. Lastly, although our individual-based approach identified interactions between comorbidities and anemia, the omission of multilevel analysis might lead to ecological fallacy. We suggest future research focuses on including interactions between individual and ecological-level factors to better understand population-level determinants. Finally, because this is an individual analysis based on cross-sectional surveys, our study was limited in deriving conclusions about the direction of causality. Hence, our results should be interpreted with caution.

## Conclusions

Using data from 27 countries and including more than 198,000 participants, this study examined potential determinants and the geospatial structure of anemia in women of reproductive age across SSA. Several interventions could be derived from this study. For poverty-related determinants, policymakers' efforts should focus on scaling up anemia screening and parasitic infections treatments to identify and reduce the likelihood of an anemia diagnosis in high-risk communities, especially women. Also, additional efforts from local governments are required to ensure that vulnerable high-risk subpopulations are not left behind within the health-related SDGs (2nd Zero hunger, 6th Clean water and sanitation). For co-occurrent epidemics and their spatial distribution in Africa, our study results highlight the additional challenges that comorbidities can pose to women at risk of anemia in SSA. We suggest multi-disease interventions in locations characterized by high comorbidity scenarios to improve health outcomes. Knowledge about the contribution of known major drivers and the spatial distribution of anemia risk can mitigate operational constraints and help to the design of geographically targeted programs in SSA. Future research needs to focus on understanding the causes and consequences of other important geographical disparities such as comparisons between urban and rural areas for all population groups at different scales.

## Supplementary Information

Supplementary Information.

## Data Availability

Data are available in a public, open-access repository. The data that support the findings of this study are available from the Demographic and Health Surveys (http://www.measuredhs.com). We sought and were granted permission to use the core data set for this analysis by Measure DHS.
