# Supplementary material for: The epidemiological landscape of anemia in women of reproductive age in sub-Saharan Africa
Source: Sci Rep. 2021 Jun 7;11:11955. doi: 10.1038/s41598-021-91198-z (PMC8184956; doi:10.1038/s41598-021-91198-z)
Supplement: Supplementary file 1 — Supplementary Information. [file 41598_2021_91198_MOESM1_ESM.pdf]

SUPLEMENTARY MATERIALS FOR:

The epidemiological landscape of anemia in women of reproductive age in sub-Saharan Africa

Esteban Correa-Agudelo, Hae-Young Kim, Godfrey N. Musuka, Zindoga Mukandavire, F. DeWolfe Miller, Frank Tanser, Diego F. Cuadros*

Reprints or correspondence: Diego F. Cuadros, Ph.D., E-mail: diego.cuadros@uc.edu, Health Geography and Disease Modeling Laboratory, University of Cincinnati, Cincinnati, OH, 45221. Telephone:(513) 556-3423. Fax: (513) 556-3370.

APPENDIX A: Covariate selection criteria and definitions

Our study used observational data from the population-based Demographic Health Survey (DHS) conducted between 2010 and 2019 in 27 countries in SSA. DHS surveys are based on cross-sectional representative samples of individuals and household level evaluation indicators on health, nutrition, and HIV serostatus over time. Moreover, DHS survey designs are standardized two-stage sampling procedures through a set of defined survey locations (primary sample units or PSU) statistically weighted to control for sample biases[1]. DHS surveys include a sample of women who had written informed consent and had a valid anemia test. Table S1 shows specific sample size by SSA country.

For this study, we estimate the relationship between anemia and the potential risk factors at the ecological-level. Ecologic studies assess the overall frequency of disease in a population for a fixed unit of analysis to make it time or geographically comparable. Therefore, the unit of observation was the number of women who tested positive for anemia at first administrative areas (Admin1), which are also referred as provinces or regions.

Figure S1 describes the sources of sociodemographic, concomitant comorbidities, and the spatial explicit cofactors according to the evidence synthesis process of relevant references. Oobservational data for reproductive women (15-49) were derived from the most recent standard and continuous Demographic Health Survey data (DHS) conducted between 2010 and 2020 in 27 in Africa (See. Table S1). Selection of covariates were carried out in several steps. For the population survey datasets, the following individual covariates were initially extracted, analyzed for completeness, and encoded properly. Further details related to each study design and survey questionnaire can be found in the DHS-7 standard recode[2-29].

Variables about reproduction Section W11 (REC11)

- V102: Type of place of residence where the respondent was interviewed as either *urban* or *rural*.
- V106: *Highest education level attended* including the following categories: *No education*, *Primary*, *Secondary*, and *Higher*.
- V113: *"Main source of drinking water for members of the household"*.

Sources of water are grouped using the World Health Organization (WHO) definition [30] (See table S1).

- V190: *"Wealth index combined"* quintile. The wealth index is a composite measure of a household's cumulative living standard. The index has been designed for easy-to-collect data on a household's ownership of selected goods, such as televisions and bicycles; materials used for housing construction; and types of water access and sanitation facilities. The quintile classification includes: *poorest*, *poorer*, *middle*, *richer*, and *richest*.

Variables about reproduction Section W22 (REC22)

- V201: "*Total number of children ever born":* Numerical
- V208: *"Total number of births in the last five years":* Numerical
- V213: *"Whether the respondent is currently pregnant"*: *yes* or *no*.

Variables about the maternity history Section W41 (REC41)

- M45: *"During pregnancy, given or bought iron tablets/syrup Days"*: *yes* or *no*.
- M54: *"Received Vitamin A dose in first 2 months after delivery"*: *yes* or *no*.
- M60: *"Drugs for intestinal parasites"* for last births in the three/five years before the survey: *yes* or *no*.

Variables about the maternity and feeding Section W42 (REC42)

- V445: *"Body mass index (BMI)"* for the respondent is defined as her weight in kilograms divided by the square of her height in meters (W/H2)· <18·5 for *underweight*, < 25·0 for *normal* weight, and >25·0 *overweight/obese*.
- V463A: *"Tobacco use (cigarettes)"*: *yes* or *no*.
- V472: *Feeding practices for the respondent.*

Variables about partner's characteristics and women's work Section W42 (REC42)

- V717: Standardized respondent's occupation groups.

The final socioeconomic and demographic individual covariates which were aggregated at admin1 level included:

- Current pregnant (V213)
- No formal or primary schooling (V106)
- Low wealth index (V190),
- Smoking cigarettes (V463A)
- Living in rural areas (V102)
- No access to improved sources of water (V113)
- Underweight (V445)
- Iron supplementation during pregnancy (M45)
- Deworming during pregnancy (M60)

For the pixel-based covariates, we obtained mean surface estimates, including 2015 malaria (*Plasmodium falciparum* incidence rate [*Pf*IR]), and 2015 HIV prevalence[31,32]. Of note the concern about the iron intake in the HIV replication for patients with both conditions[33]. These continuous surfaces were generated through model-based geostatistical approach at a fixed grid resolution by combining surveys and clinical data sources. Similarly, inequality in accessibility was assessed as spatial covariate using the predicted travel time in hours to nearest city[34]. Then,

Figure S1. Study area and survey sites (black dots).

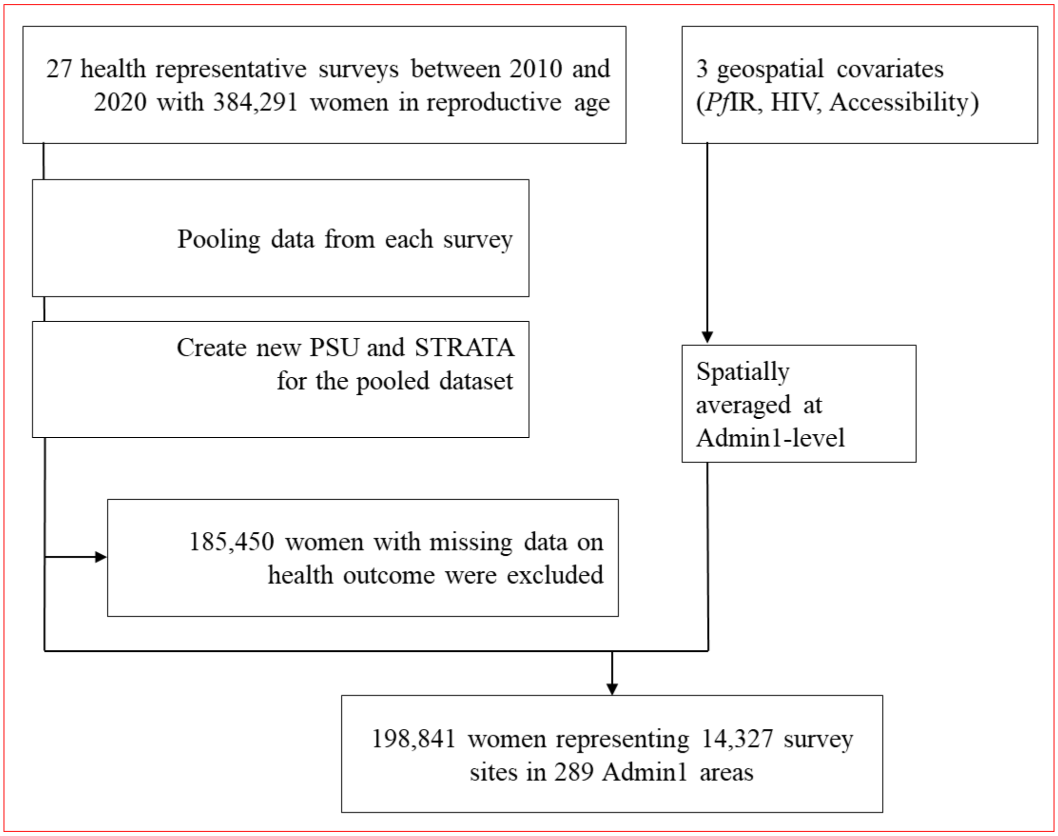

Table S1. List of analyzed countries and sample size.

Country	ISO alpha-3	Sample size
Burundi	BDI	8,587
Benin	BEN	8,018
Burkina Faso	BFA	8,424
Ivory Coast	CIV	4,589
Cameroon	CMR	7,803
Democratic Republic of Congo	COD	9,349
Congo	COG	5,364
Ethiopia	ETH	14,923
The Gabon	GAB	5,267
Ghana	GHA	4,644
Guinea	GIN	5,217
Gambia	GMB	4,489
Lesotho	LSO	3,297
Mali	MLI	5,063
Mozambique	MOZ	13,571
Malawi	MWI	7,933
Namibia	NAM	4,242
Niger	NER	5,060
Nigeria	NGA	14,617
Rwanda	RWA	6,680
Senegal	SEN	7,906
Sierra Leone	SLE	7,869
Togo	TGO	4,782
Tanzania	TZA	13,064
Uganda	UGA	5,988
South Africa	ZAF	2,857
Zimbabwe	ZWE	9236

Table S2. Water recodes.

Original survey codification	Type of water source
"piped water",<br>"piped into dwelling",<br>"piped to yard/plot",<br>"piped to neighbor",<br>"other piped",<br>"public tap/standpipe",<br>"tube well water",<br>"tube well or borehole",<br>"dug well (open/protected)",<br>"protected well",<br>"mechanical or manual well",<br>"protected spring",<br>"rainwater",<br>"bottled water",<br>"water bags",<br>"sachet water",<br>"bag water",<br>"water vendor",<br>"water in plastic bag",<br>"neighbor’s tap",<br>"piped from the neighbor",<br>"borehole with pump",<br>"neighbor's house"	Improved drinking-water sources
"unprotected well",<br>"surface water",<br>"unprotected spring",	Unimproved drinking-water sources

"river/dam/lake/ponds/stream/canal/irrigation channel",	
"river/irrigation channel",	
"lake or stream",	
"tanker truck",	
"cart with small tank",	
"public fountain/tank",	
"bicycle with jerrycans",	
"other",	
"community plant",	

APPENDIX B: Statistical Analysis

We stack population surveys from each country into one full dataset. In order to include the two-stage sampling procedure while preserving correct sampling error estimates, new PSU and STRATA were built combining country identification and previous survey design information into the full dataset. All covariates are included into a logistic model for complex surveys including the estimation of the variance inflation factors (VIF) as a test of multicollinearity [35] (See Table S3). First, we stack population surveys from each country into one full dataset. In order to include the two-stage sampling procedure while preserving correct sampling error estimates, new PSU and STRATA were built combining country identification and previous survey design information into the full dataset. Finally, an additional verification step was performed to verify the correctness of our full dataset. We compared the official DHS estimates for anemia prevalence (https://www.statcompiler.com/en/) with our current estimates using the final samples at national and admin1-level (See Fig. S2). Models were fitted using the integrated nested Laplace approximation (INLA) Bayesian framework. INLA is a faster approach and a valid alternative to the commonly used Markov Chain Monte Carlo (MCMC) simulations for Bayesian models [36].All statistical analyses including unadjusted and adjusted health survey's models, were conducted in R programming environment and *Survey* package [37,38].

Table S3. Variance inflated factor (VIF) model selection results.

	**VIF score**
**Age-group**	
15-24	Ref
25-34	1·10
35-44	1·10
45+	1·10
**Type of place**	
Urban	Ref
Rural	1·58
**Education**	
Higher	Ref
Primary/Secondary	1·27

No education	1·27
**Source of drinking water**	
Improved source	Ref
Unimproved source	1·13
**Wealth Index**	
Richest	Ref
Richer	1·80
Middle	1·80
Poorer	1·80
Poorest	1·80
**BMI**	
Normal/Obese	Ref
Underweight	1·05
**Smoking cigarettes**	
No	Ref
Yes	1·01
**Current Pregnant**	
No or unsure	Ref
Yes	1·01
**Iron supplementation during pregnancy**	
No	Ref
Yes	1·07
**Deworming during pregnancy**	
No	Ref
Yes	1·10
**Admin-1 level**	
PfIR	1·31
HIV prevalence	1·33
Accessibility to main cities (Hour)	1·03

Figure S2. Estimated anemia prevalence in SSA.

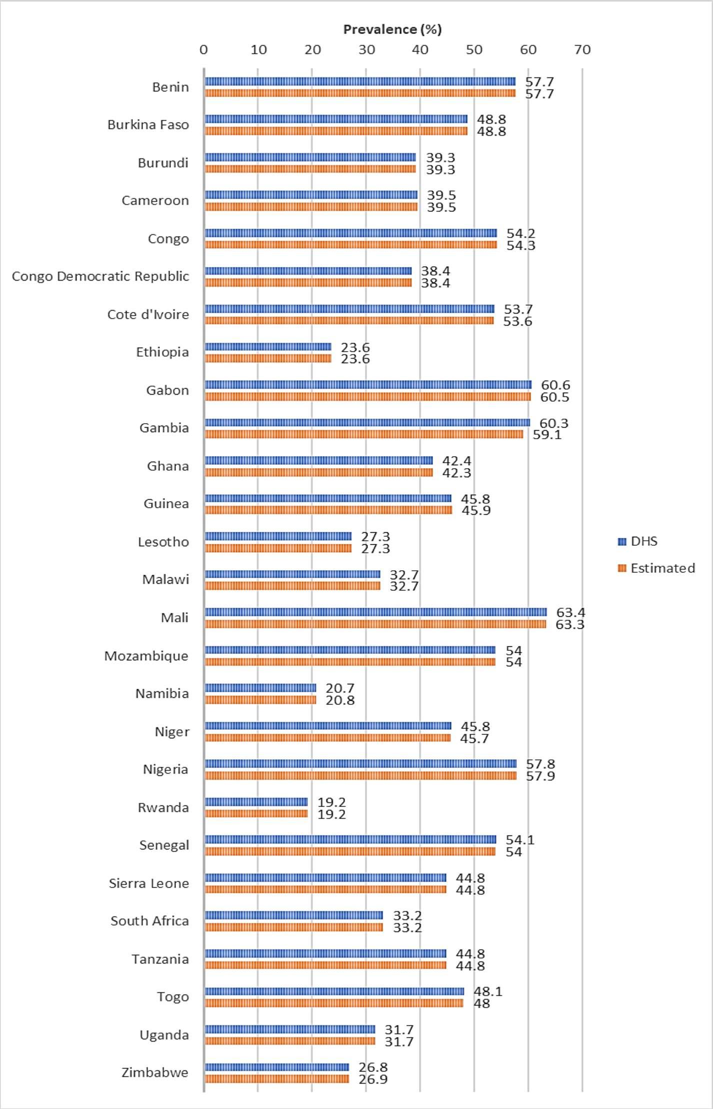

Table S4. Unadjusted odds ratios showing associations between anemia and control variables.

	OR (95% CI)
**Age-group**	
15-24	Ref
25-34	**0·96 (0·93 , 0·98)**
35-44	1·02 (0·99 , 1·05)
45+	0·98 (0·94 , 1·02)
**Type of place**	
Urban	Ref

Rural	0·97	(0·94	,	1·00)
**Education**				
Higher	Ref			
Primary/Secondary	**1·21**	**(1·12**	**,**	**1·29)**
No education	**1·72**	**(1·59**	**,**	**1·85)**
**Type of water**				
Improved source	Ref			
Unimproved source	**1·12**	**(1·08**	**,**	**1·16)**
**Wealth Index**				
Richest	Ref			
Richer	**1·12**	**(1·08**	**,**	**1·17)**
Middle	**1·23**	**(1·18**	**,**	**1·29)**
Poorer	**1·26**	**(1·21**	**,**	**1·32)**
Poorest	**1·14**	**(1·31**	**,**	**1·43)**
**BMI**				
Normal/Obese	Ref			
Underweight	**1·15**	**(1·11**	**,**	**1·19)**
**Smoking cigarettes**				
No	Ref			
Yes	0·93	(0·82	,	1·06)
**Current pregnant**				
No or unsure	Ref			
Yes	**1·43**	**(1·38**	**,**	**1·49)**
**Iron supplementation during pregnancy**				
No	Ref			
Yes	0·94	(0·94	,	1·02)
**Deworming during pregnancy**				
No	Ref			
Yes	**1·07**	**(1·03**	**,**	**1·10)**
**Admin-1 level**				
PfIR	**1·02**	**(1·02**	**,**	**1·02)**

HIV prevalence	**0·98**	**(0·98**	**,**	**0·99)**
Accessibility to main cities (Hour)	1·00	(0·99	,	1·00)

Notes: Boldfaced numbers indicate statistical association <0·05.

Abbreviations: OR, odds ratio; CI, confidence interval.

Table S5. Descriptive statistics by country.

	BDI	BEN	BFA	CIV	CMR	COD	COG	ETH	GAB	GHA	GIN	GMB	LSO	MLI	MOZ
**Age-group**															
15-24	41·4	39·0	38·8	40·9	43·9	40·7	39·9	38·8	39·8	34·5	40·7	44·2	42·2	37·0	40·2
25-34	31·7	32·5	33·0	33·1	30·3	32·8	33·1	34·1	32·1	31·7	30·5	33·0	31·0	35·1	31·0
35-44	20·1	20·3	20·7	19·2	18·3	19·8	20·1	20·5	21·0	24·8	19·9	17·6	19·1	21·7	20·8
45+	6·9	8·2	7·6	6·9	7·5	6·7	6·9	6·6	7·1	9·0	8·8	5·2	7·7	6·3	8·0
**Type of place**															
Urban	13·1	41·6	26·5	49·7	53·8	36·7	66·4	21·2	88·2	53·9	36·2	54·4	34·6	25·6	34·2
Rural	86·9	58·4	73·5	50·3	46·2	63·3	33·6	78·8	11·8	46·1	63·8	45·6	65·4	74·4	65·8
**Education**															
No education	35·7	55·5	74·1	54·7	19·1	15·9	5·8	48·3	5·1	19·5	68·2	47·2	1·1	66·8	31·4
Primary	39·2	19·4	13·8	25·5	34·7	37·2	25·7	35·1	22·2	18·2	12·3	13·8	39·3	13·2	50·3
Secondary	23·8	23·2	11·0	17·7	40·6	43·4	64·1	11·2	63·7	56·6	15·8	34·0	51·6	18·1	17·0
Higher	1·3	1·8	1·0	2·2	5·5	3·4	4·4	5·3	9·0	5·8	3·7	5·0	7·9	1·9	1·3
Missing data	0·0	0·0	0·1	0·0	0·0	0·0	0·0	0·0	0·0	0·0	0·0	0·0	0·0	0·0	0·0
**Source of drinking water**															
Improved source	82·6	69·2	78·1	78·6	69·9	51·0	73·1	64·1	89·3	87·3	79·8	89·9	79·9	70·4	54·3
Unimproved source	16·7	28·5	20·9	19·7	26·0	47·2	20·8	33·5	7·3	10·1	18·8	8·9	14·5	28·9	44·0
Missing data	0·7	2·3	1·0	1·6	4·1	1·8	6·1	2·4	3·4	2·6	1·4	1·2	5·6	0·7	1·6
**Wealth Index**															
Richest	21·1	22·9	24·2	25·0	24·0	22·8	19·8	25·6	21·0	22·8	22·2	23·9	25·5	22·2	23·2
Richer	20·2	22·2	19·5	20·0	23·5	19·5	19·9	20·0	22·1	22·1	18·8	21·2	24·2	21·1	20·2

Middle	20·0	20·7	18·9	18·1	18·6	19·6	21·1	19·4	22·4	20·9	19·0	17·3	19·0	19·2	18·8
Poorer	19·8	18·1	19·1	18·5	18·0	19·1	21·2	18·2	19·3	17·2	20·5	20·2	16·8	20·4	18·7
Poorest	18·9	16·1	18·3	18·3	15·9	19·1	17·9	16·9	15·2	17·0	19·5	17·3	14·6	17·1	19·0
**BMI**															
Normal/Obese	82·3	89·8	85·2	92·0	93·2	86·6	86·5	78·8	93·3	94·1	90·5	82·7	95·8	90·5	92·1
Underweight	17·6	10·1	14·8	7·0	6·6	13·1	13·5	21·1	6·7	5·7	9·1	15·1	4·1	9·4	7·9
Missing data	0·1	0·1	0·0	1·0	0·1	0·3	0·0	0·1	0·0	0·2	0·4	2·2	0·2	0·1	0·0
**Smoking cigarettes**															
No	99·2	98·6	99·8	99·5	0·0	99·5	99·3	99·4	97·1	99·9	99·1	99·7	99·8	99·3	98·6
Yes	0·8	1·4	0·1	0·3	0·0	0·5	0·6	0·6	2·8	0·1	0·9	0·2	0·2	0·7	1·4
Missing data	0·0	0·0	0·0	0·2	100·0	0·0	0·1	0·0	0·1	0·0	0·0	0·1	0·0	0·0	0·0
**Current Pregnant**															
No or unsure	91·9	89·1	89·8	89·4	90·4	88·0	91·1	92·7	89·9	92·7	91·3	92·1	95·8	88·5	88·9
Yes	8·1	10·9	10·2	10·6	9·6	12·0	8·9	7·3	10·1	7·3	8·7	7·9	4·2	11·5	11·1
**Iron supplementation during pregnancy**															
No	27·1	8·0	0·0	12·0	9·3	24·5	0·0	28·2	0·0	3·3	9·9	1·8	9·0	14·5	10·9
Yes	24·7	48·4	0·0	41·5	40·9	34·6	0·0	20·8	0·0	41·3	42·3	51·2	29·8	48·9	46·5
Missing data	48·2	43·5	100·0	46·6	49·9	40·9	100·0	51·0	100·0	55·4	47·7	47·0	61·1	36·6	42·7
**Deworming during pregnancy**															
No	17·1	18·2	45·6	32·4	29·9	26·1	7·8	45·6	12·1	26·2	33·9	29·6	0·0	30·7	39·5
Yes	34·7	36·9	16·0	20·3	18·8	32·9	48·0	2·9	32·8	18·0	17·7	21·8	0·0	31·1	16·2

Missing data	48·2	44·9	38·4	47·3	51·2	41·0	44·2	51·5	55·1	55·8	48·5	48·6	100·0	38·2	44·4

Table S5 (continued)

	MWI	NAM	NER	NGA	RWA	SEN	SLE	TGO	TZA	UGA	ZAF	ZWE	SSA Mean	SSA Std
**Age-group**														
15-24	42·9	40·4	34·3	34·2	39·1	40·7	38·0	36·2	40·7	43·2	33·3	39·4	39·4	2·9
25-34	31·0	29·6	36·7	33·9	32·6	32·8	31·6	31·7	29·3	30·4	32·3	33·0	32·2	1·6
35-44	19·8	22·2	21·7	23·3	21·1	20·3	22·2	23·8	22·5	20·1	24·0	21·9	21·0	1·7
45+	6·3	7·8	7·3	8·5	7·2	6·2	8·1	8·3	7·5	6·2	10·4	5·7	7·4	1·1
**Type of place**														
Urban	17·8	54·3	17·8	46·4	19·8	49·2	35·9	45·1	35·8	25·8	62·4	37·5	40·1	17·3
Rural	82·2	45·7	82·2	53·6	80·2	50·8	64·1	54·9	64·2	74·2	37·6	62·5	59·9	17·3
**Education**														
No education	12·0	4·8	80·5	33·0	11·9	45·5	56·1	31·3	14·6	9·7	2·2	1·3	31·5	24·9
Primary	61·3	20·2	11·5	15·2	64·6	24·0	13·9	34·4	62·1	57·4	10·9	25·7	29·7	16·9
Secondary	23·6	66·5	7·4	41·4	20·7	25·7	27·0	31·1	22·0	25·6	77·3	66·0	34·3	20·1
Higher	3·0	8·5	0·5	10·3	2·8	4·8	3·0	3·2	1·2	7·2	9·7	6·9	4·5	2·9
Missing data	0·0	0·0	0·1	0·0	0·0	0·0	0·0	0·0	0·0	0·0	0·0	0·0	0·0	0·0
**Source of drinking water**														
Improved source	87·2	83·5	66·8	70·9	73·2	75·5	60·3	67·8	60·2	76·7	88·7	75·7	74·2	10·5
Unimproved source	12·0	13·4	32·9	27·9	25·1	20·4	39·0	29·8	34·5	19·4	9·1	19·9	23·3	10·7
Missing data	0·8	3·1	0·3	1·2	1·7	4·0	0·7	2·4	5·3	3·9	2·3	4·4	2·5	1·6

**Wealth Index**														
Richest	23·2	22·1	21·2	22·6	23·3	24·8	24·0	24·5	26·8	25·1	14·9	24·3	23·1	2·3
Richer	18·9	24·1	21·4	22·0	18·8	20·1	19·6	23·5	21·3	20·4	18·9	23·2	21·0	1·7
Middle	18·9	19·2	20·6	20·4	18·7	20·3	19·2	18·3	17·7	18·7	23·1	18·0	19·5	1·4
Poorer	19·9	18·4	18·9	18·8	19·7	18·0	19·1	16·3	17·3	17·8	19·9	17·2	18·8	1·2
Poorest	19·2	16·2	17·8	16·1	19·6	16·7	18·0	17·4	17·0	18·0	23·2	17·3	17·7	1·7
**BMI**														
Normal/Obese	93·2	86·6	86·3	88·6	93·8	0·0	91·5	93·3	91·2	91·7	96·2	94·0	86·7	17·9
Underweight	6·7	13·0	13·7	11·3	6·1	0·0	8·5	6·4	8·7	8·0	2·9	5·8	9·4	4·7
Missing data	0·1	0·4	0·0	0·1	0·1	100·0	0·0	0·2	0·1	0·3	0·8	0·3	4·0	19·2
**Smoking cigarettes**														
No	99·4	96·0	99·9	99·8	99·5	99·4	95·2	99·9	99·6	99·2	95·9	99·7	95·3	19·1
Yes	0·6	3·9	0·0	0·2	0·5	0·6	4·7	0·1	0·4	0·8	4·1	0·3	1·0	1·3
Missing data	0·0	0·1	0·1	0·0	0·0	0·0	0·1	0·0	0·0	0·0	0·0	0·0	3·7	19·2
**Current Pregnant**														
No or unsure	91·9	93·2	85·3	89·5	92·7	91·8	91·5	91·1	91·4	89·7	96·3	93·7	91·1	2·3
Yes	8·1	6·8	14·7	10·5	7·3	8·2	8·5	8·9	8·6	10·3	3·7	6·3	8·9	2·3
**Iron supplementation during pregnancy**														
No	6·1	4·9	0·0	16·2	9·4	1·4	0·0	6·8	10·1	6·6	3·6	7·1	8·5	8·0
Yes	50·1	37·3	0·0	41·5	35·1	46·7	0·0	44·3	43·4	49·2	34·3	43·2	33·2	17·7
Missing data	43·8	57·8	100·0	42·3	55·5	51·9	100·0	49·0	46·5	44·2	62·0	49·7	58·3	21·1

**Deworming during pregnancy**														
No	26·4	37·7	34·2	46·5	22·6	26·3	12·9	21·0	19·5	23·2	0·0	48·3	26·4	13·2
Yes	29·4	2·9	36·2	10·5	21·9	19·1	39·1	28·9	33·7	32·2	0·0	1·7	22·4	13·3
Missing data	44·2	59·3	29·6	43·1	55·5	54·6	47·9	50·2	46·8	44·6	100·0	50·0	51·2	15·4

APPENDIX C: Disease mapping

Disease mapping is the process of visually depicting geographically indexed data in a spatial referenced distribution for explanatory purposes. In spatial statistical methods, small area disease models are commonly used to quantify risk estimates and spatial autocorrelation using areal data. Benefits from the model-based disease mapping for this study are two-fold [39]: first, they allow to introduce a reference population to obtain reliable risk estimates of a disease based on Standardized Incidence Ratios (ratio of the observed to the expected disease counts [SIRs]). Second, disease models offer a smoothing mechanism to improve local risk estimates while avoiding extreme values of areas with small populations when incorporate mixed effects and spatial dependence (relative risk [RR]).

The Besag-York-Mollie model in Small area disease models is expressed as

$$Y_i \sim Po(E_i \times \theta_i), i = 1, \dots, n$$

$$\log(\theta_i) = \alpha + \sigma_i + v_i$$

Where α denotes the overall risk level, σ_i is the structured spatial effect that models the dependence of neighbors' relative risk, and v_i is the uncorrelated variance.

R programming environment (Version 3.6.3; https://cran.r-project.org/bin/windows/base/old/3.6.3/) and packages including *INLA* [36,40], *SpatialEpi* [41], *ggplot2* and *raster* were used to generate all maps.

Table S6. Relative risk by Admin1.

Region	Location	Anemia Prevalence (%)	Pregnancy (%)	No formal or primary schooling (%)	Poverty (%)	Smokes cigarettes (%)	Living in rural area (%)	Accessibility to main cities (Hour)
pointe-noire	COG, Central Africa	0·60	0·09	0·25	0·00	0·01	0·00	0·01
libreville-port-gentil	GAB, Central Africa	0·62	0·10	0·20	0·01	0·03	0·00	0·06
banjul	GMB, Western Africa	0·52	0·07	0·37	0·00	0·00	0·00	0·22
bas-congo	COD, Central Africa	0·55	0·11	0·50	0·06	0·00	0·71	2·69
bandundu	COD, Central Africa	0·38	0·13	0·49	0·22	0·00	0·79	7·96
centre-nord	CIV, Western Africa	0·75	0·09	0·83	0·15	0·00	0·45	1·68
niamey	NER, Western Africa	0·47	0·12	0·67	0·00	0·00	0·00	0·19
kinshasa	COD, Central Africa	0·47	0·05	0·10	0·00	0·00	0·00	2·16
acholi	UGA, Eastern Africa	0·47	0·11	0·75	0·55	0·01	0·77	1·58
caprivi	NAM, Southern Africa	0·27	0·05	0·23	0·28	0·02	0·66	4·74
sahel	BFA, Western Africa	0·69	0·13	0·98	0·49	0·00	0·95	2·32
kaskazinipemba	TZA, Eastern Africa	0·71	0·13	0·57	0·02	0·00	0·82	3·76

lome	TGO, Western Africa	0·56	0·07	0·51	0·00	0·00	0·00	0·01
janjanbureh	GMB, Western Africa	0·74	0·08	0·73	0·32	0·00	0·81	2·47
shinyanga	TZA, Eastern Africa	0·60	0·10	0·85	0·33	0·01	0·73	1·72
dosso	NER, Western Africa	0·58	0·12	0·94	0·12	0·00	0·90	2·33
maseru	LSO, Southern Africa	0·34	0·04	0·33	0·04	0·00	0·42	1·14
northwest	ZAF, Southern Africa	0·41	0·05	0·18	0·12	0·03	0·60	1·96
maniema	COD, Central Africa	0·50	0·13	0·58	0·22	0·01	0·68	7·83
mjinimagharibi	TZA, Eastern Africa	0·58	0·06	0·22	0·00	0·01	0·48	0·11
sofala	MOZ, Eastern Africa	0·58	0·10	0·82	0·14	0·01	0·56	2·62
sud-ouest	CMR, Central Africa	0·54	0·07	0·45	0·01	0·00	0·48	3·34
littoral	CMR, Central Africa	0·38	0·08	0·36	0·03	0·00	0·35	3·21
bulawayo	ZWE, Eastern Africa	0·29	0·04	0·09	0·00	0·01	0·00	0·07
kavango	NAM, Southern Africa	0·33	0·08	0·49	0·44	0·04	0·71	7·87

mpumalanga	ZAF, Southern Africa	0·38	0·03	0·13	0·24	0·03	0·59	0·93
mansakonko	GMB, Western Africa	0·67	0·08	0·67	0·39	0·01	0·83	1·29
maputocidade	MOZ, Eastern Africa	0·49	0·06	0·45	0·00	0·01	0·00	0·39
matabelelandsouth	ZWE, Eastern Africa	0·43	0·06	0·30	0·19	0·01	0·82	2·57
kirundo	BDI, Eastern Africa	0·60	0·10	0·88	0·37	0·00	0·95	0·49
mamou	GIN, Western Africa	0·46	0·07	0·90	0·17	0·00	0·85	1·56
mwanza	TZA, Eastern Africa	0·55	0·11	0·77	0·17	0·01	0·52	2·10
ruyigi	BDI, Eastern Africa	0·54	0·09	0·86	0·28	0·00	0·95	0·58
douala	CMR, Central Africa	0·53	0·09	0·21	0·00	0·00	0·00	1·57
lindi	TZA, Eastern Africa	0·49	0·07	0·87	0·18	0·01	0·77	8·74
bouenza	COG, Central Africa	0·50	0·08	0·53	0·27	0·00	0·66	2·75
ngozi	BDI, Eastern Africa	0·48	0·06	0·82	0·34	0·01	0·92	0·35
cankuzo	BDI, Eastern Africa	0·54	0·10	0·87	0·30	0·00	0·98	1·12
alibori	BEN, Western Africa	0·58	0·12	0·91	0·23	0·06	0·76	2·41
kayes	MLI, Western Africa	0·73	0·12	0·88	0·10	0·01	0·84	3·89
tabora	TZA, Eastern Africa	0·53	0·13	0·89	0·41	0·00	0·82	4·24

daressalaam	TZA, Eastern Africa	0·53	0·06	0·57	0·00	0·01	0·00	0·36
greateraccra	GHA, Western Africa	0·42	0·07	0·25	0·01	0·00	0·10	0·45
lango	UGA, Eastern Africa	0·39	0·12	0·86	0·42	0·00	0·93	1·44
cibitoke	BDI, Eastern Africa	0·52	0·09	0·81	0·14	0·01	0·95	1·07
southeast	NGA, Western Africa	0·66	0·09	0·23	0·04	0·00	0·29	0·30
centre	CMR, Central Africa	0·48	0·09	0·43	0·02	0·00	0·65	3·18
basse	GMB, Western Africa	0·72	0·11	0·90	0·16	0·00	0·92	2·16
brazzaville	COG, Central Africa	0·54	0·06	0·16	0·00	0·00	0·00	0·08
mtwara	TZA, Eastern Africa	0·47	0·07	0·87	0·17	0·01	0·74	3·98
inhambane	MOZ, Eastern Africa	0·57	0·08	0·81	0·04	0·00	0·71	3·17
western	GHA, Western Africa	0·43	0·07	0·34	0·05	0·00	0·57	2·64
kasai-oriental	COD, Central Africa	0·41	0·13	0·52	0·18	0·00	0·60	9·37
simiyu	TZA, Eastern Africa	0·54	0·09	0·83	0·35	0·01	0·96	3·96
harare	ZWE, Eastern Africa	0·30	0·06	0·09	0·00	0·01	0·06	0·02
northern	SLE, Western Africa	0·50	0·09	0·77	0·17	0·05	0·84	2·22

dakar	SEN, Western Africa	0·53	0·06	0·53	0·00	0·01	0·03	0·14
midlands	ZWE, Eastern Africa	0·31	0·06	0·28	0·22	0·00	0·70	1·88
atacora	BEN, Western Africa	0·67	0·10	0·81	0·39	0·00	0·68	2·23
central	GHA, Western Africa	0·47	0·07	0·30	0·04	0·00	0·61	0·88
boucledemouhoun	BFA, Western Africa	0·49	0·11	0·94	0·11	0·00	0·91	1·72
kuntaur	GMB, Western Africa	0·73	0·12	0·85	0·35	0·00	0·94	2·33
plateau	BEN, Western Africa	0·71	0·13	0·80	0·19	0·03	0·56	0·83
mara	TZA, Eastern Africa	0·51	0·12	0·79	0·22	0·00	0·75	3·02
south	RWA, Eastern Africa	0·23	0·07	0·81	0·26	0·01	0·90	1·04
equateur	COD, Central Africa	0·35	0·15	0·65	0·39	0·00	0·82	8·80
southernregion	MWI, Eastern Africa	0·36	0·08	0·75	0·19	0·01	0·83	1·36
busoga	UGA, Eastern Africa	0·41	0·13	0·60	0·12	0·01	0·85	2·39
tanga	TZA, Eastern Africa	0·44	0·07	0·74	0·09	0·00	0·59	2·95

tete	MOZ, Eastern Africa	0·54	0·11	0·88	0·24	0·03	0·88	4·45
kindia	GIN, Western Africa	0·57	0·08	0·84	0·20	0·00	0·63	1·38
northeast	NGA, Western Africa	0·58	0·12	0·71	0·34	0·00	0·69	1·51
cabodelgado	MOZ, Eastern Africa	0·62	0·12	0·92	0·26	0·03	0·85	3·81
oromia	ETH, Eastern Africa	0·27	0·08	0·88	0·15	0·01	0·85	3·03
zambezia	MOZ, Eastern Africa	0·62	0·14	0·93	0·43	0·03	0·84	1·81
northwest	NGA, Western Africa	0·59	0·14	0·76	0·28	0·00	0·70	1·03

Table S4 (continued)

**Region**	**Unimproved source of water (%)**	**Women in Underweight (%)**	***Pf*IR (%)**	**HIV Prevalence (%)**	**Anemia RR CrI: [2.5%, 97.5%]**
pointe-noire	0·03	0·12	0·20	3·53	7·05 [6·59, 7·53]
libreville-port-gentil	0·02	0·06	0·24	3·88	4·35 [4·15, 4·55]
banjul	0·01	0·11	0·02	1·62	3·93 [2·89, 5·13]
bas-congo	0·36	0·22	0·26	0·47	1·98 [1·74, 2·23]
bandundu	0·69	0·24	0·21	0·43	1·86 [1·71, 2·01]

centre-nord	0·13	0·06	0·44	3·37	1·82 [1·61, 2·03]
niamey	0·03	0·08	0·20	0·34	1·7 [1·47, 1·94]
kinshasa	0·01	0·07	0·31	0·40	1·68 [1·53, 1·83]
acholi	0·20	0·14	0·25	6·63	1·56 [1·32, 1·83]
caprivi	0·13	0·11	0·05	20·88	1·52 [1·16, 1·93]
sahel	0·39	0·21	0·44	0·24	1·51 [1·37, 1·66]
kaskazinipemba	0·06	0·11	0·10	0·33	1·51 [1·1, 1·98]
lome	0·06	0·04	0·15	3·69	1·49 [1·39, 1·59]
janjanbureh	0·20	0·22	0·03	1·97	1·49 [1·31, 1·68]
shinyanga	0·46	0·07	0·10	5·29	1·49 [1·32, 1·66]
dosso	0·56	0·11	0·39	0·37	1·46 [1·32, 1·61]
maseru	0·04	0·04	NA	24·43	1·45 [1·29, 1·61]
northwest	0·04	0·04	0·03	16·65	1·45 [1·18, 1·74]
maniema	0·31	0·08	0·36	0·91	1·42 [1·2, 1·66]
mjinimagharibi	0·01	0·12	0·07	0·88	1·42 [1·17, 1·68]
sofala	0·35	0·09	0·34	12·03	1·41 [1·32, 1·51]
sud-ouest	0·26	0·03	0·26	4·11	1·4 [1·25, 1·56]
littoral	0·27	0·02	0·23	3·62	1·4 [1·16, 1·65]
bulawayo	0·00	0·05	0·02	15·64	1·37 [1·17, 1·59]
kavango	0·35	0·16	0·11	13·20	1·37 [1·14, 1·61]
mpumalanga	0·09	0·04	0·09	22·09	1·36 [1·11, 1·62]
mansakonko	0·05	0·21	0·02	1·77	1·35 [1·15, 1·58]
maputocidade	0·01	0·05	0·14	18·10	1·35 [1·23, 1·49]
matabelelandsouth	0·18	0·11	0·02	19·15	1·35 [1·15, 1·55]

kirundo	0·24	0·17	0·26	1·69	1·34 [1·2, 1·48]
mamou	0·36	0·11	0·40	1·26	1·34 [1·17, 1·52]
mwanza	0·31	0·08	0·07	4·54	1·31 [1·2, 1·43]
ruyigi	0·26	0·18	0·17	0·87	1·31 [1·15, 1·48]
douala	0·01	0·03	0·21	4·01	1·31 [1·2, 1·43]
lindi	0·48	0·06	0·13	2·61	1·3 [1·1, 1·52]
bouenza	0·39	0·18	0·14	3·39	1·29 [1·15, 1·44]
ngozi	0·12	0·21	0·16	1·33	1·29 [1·16, 1·43]
cankuzo	0·33	0·18	0·21	1·03	1·29 [1·08, 1·52]
alibori	0·62	0·09	0·46	0·44	1·28 [1·17, 1·39]
kayes	0·31	0·15	0·46	1·07	1·28 [1·17, 1·38]
tabora	0·62	0·09	0·10	4·81	1·27 [1·15, 1·4]
daressalaam	0·15	0·07	0·11	4·87	1·27 [1·18, 1·36]
greateraccra	0·02	0·04	0·16	2·38	1·26 [1·14, 1·38]
lango	0·13	0·12	0·29	5·88	1·26 [1·06, 1·47]
cibitoke	0·19	0·16	0·17	1·29	1·25 [1·1, 1·42]
southeast	0·16	0·05	0·29	3·65	1·25 [1·18, 1·32]
centre	0·22	0·03	0·34	4·67	1·25 [1·1, 1·4]
basse	0·05	0·16	0·04	1·88	1·24 [1·12, 1·36]
brazzaville	0·06	0·13	0·19	2·22	1·23 [1·15, 1·31]
mtwara	0·33	0·09	0·13	3·14	1·22 [1·06, 1·4]
inhambane	0·37	0·04	0·22	15·38	1·22 [1·12, 1·33]
western	0·13	0·04	0·40	1·66	1·21 [1·06, 1·37]
kasai-oriental	0·60	0·16	0·30	0·77	1·21 [1·09, 1·33]

simiyu	0·33	0·13	0·06	3·10	1·2 [1·06, 1·35]
harare	0·03	0·03	0·03	13·26	1·2 [1·1, 1·31]
northern	0·58	0·09	0·40	1·13	1·2 [1·14, 1·26]
dakar	0·01	0·00	0·06	0·34	1·2 [1·13, 1·27]
midlands	0·29	0·07	0·06	13·82	1·2 [1·08, 1·33]
atacora	0·38	0·14	0·39	0·74	1·2 [1·09, 1·31]
central	0·10	0·04	0·37	1·80	1·19 [1·04, 1·35]
boucledemouhoun	0·34	0·13	0·50	0·65	1·19 [1·08, 1·3]
kuntaur	0·14	0·17	0·02	1·80	1·19 [1·02, 1·37]
plateau	0·22	0·15	0·27	0·76	1·19 [1·07, 1·32]
mara	0·54	0·09	0·08	2·65	1·18 [1·05, 1·33]
south	0·30	0·09	0·06	2·09	1·17 [1·06, 1·29]
equateur	0·77	0·12	0·26	0·65	1·17 [1·07, 1·28]
southernregion	0·11	0·07	0·14	11·42	1·16 [1·1, 1·22]
busoga	0·09	0·06	0·18	4·31	1·16 [1·01, 1·31]
tanga	0·25	0·08	0·12	2·31	1·15 [1·02, 1·28]
tete	0·53	0·09	0·28	5·81	1·14 [1·07, 1·22]
kindia	0·34	0·07	0·42	1·31	1·14 [1·03, 1·25]
northeast	0·44	0·22	0·36	2·94	1·14 [1·07, 1·2]
cabodelgado	0·62	0·08	0·33	10·81	1·13 [1·04, 1·22]
oromia	0·36	0·23	0·01	0·69	1·11 [1·06, 1·17]
zambezia	0·75	0·13	0·47	11·58	1·1 [1·05, 1·16]
northwest	0·43	0·15	0·45	1·70	1·07 [1·02, 1·11]

Figure S3. Anemia prevalence: A) Mild & moderate anemia prevalence, mean=42·01 (range is 14·6-73·6); B) Severe anemia prevalence, mean=0·96 (range is 0·0-8·9). Maps were generated using R programming environment, version 3.6.3 (https://cran.r-project.org/bin/windows/base/old/3.6.3/)

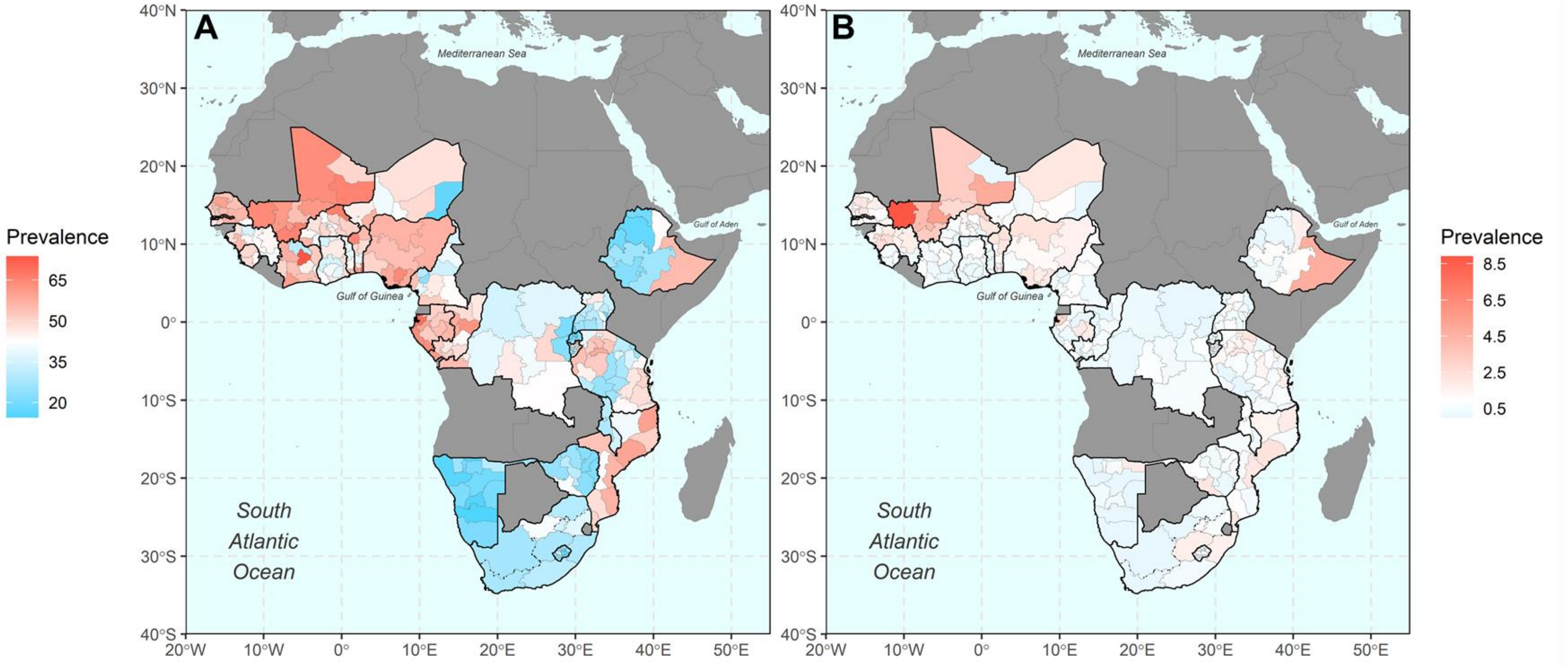

Figure S4. Sickle cell allele frequency, mean=6·1 (range is 0·1-15·2). Maps were generated using R programming environment, version 3.6.3 (https://cran.r-project.org/bin/windows/base/old/3.6.3/)

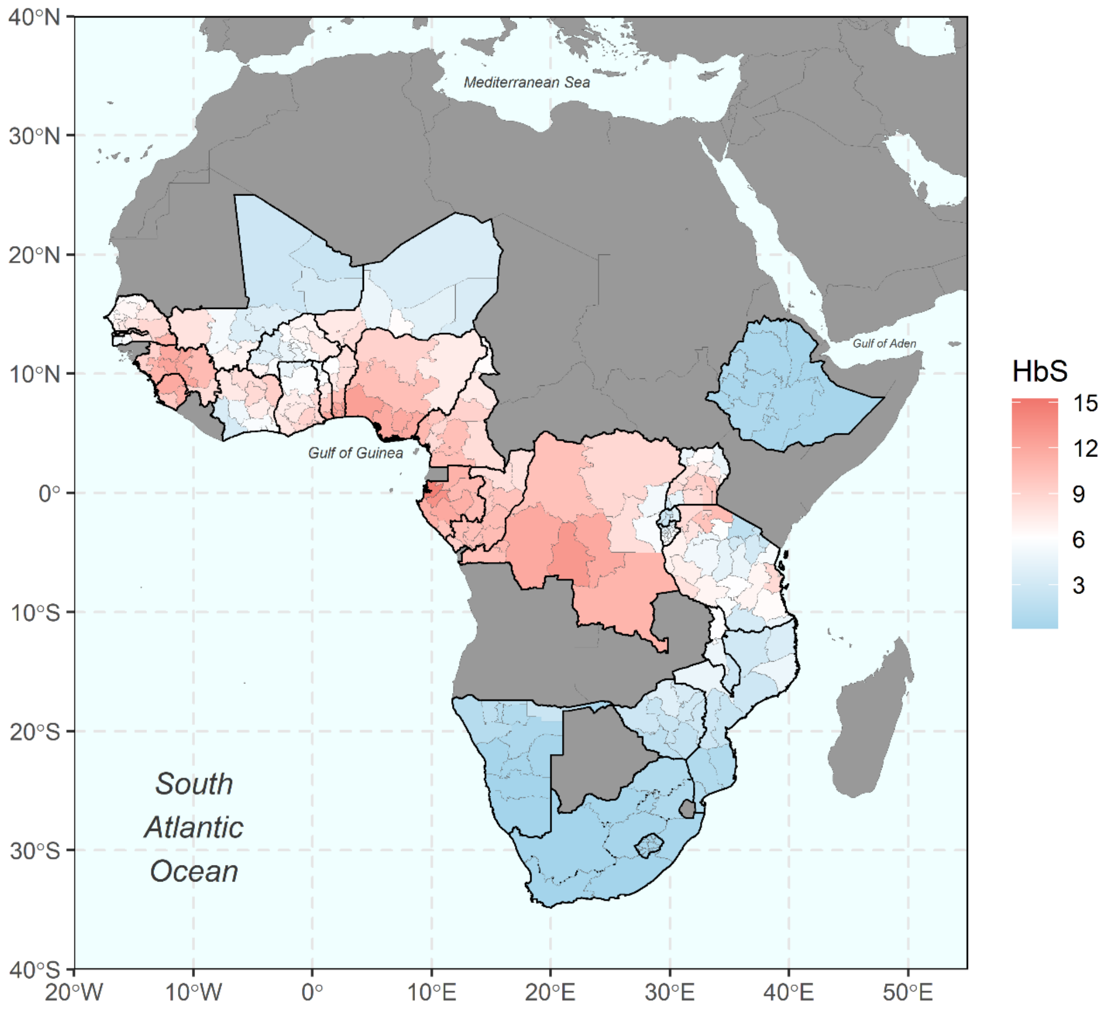

Figure S5. *Plasmodium falciparum* incidence rate (*PF*IR), mean=21·4 (range is 0··5-62·3). Maps were generated using R programming environment, version 3.6.3 (https://cran.r-project.org/bin/windows/base/old/3.6.3/)

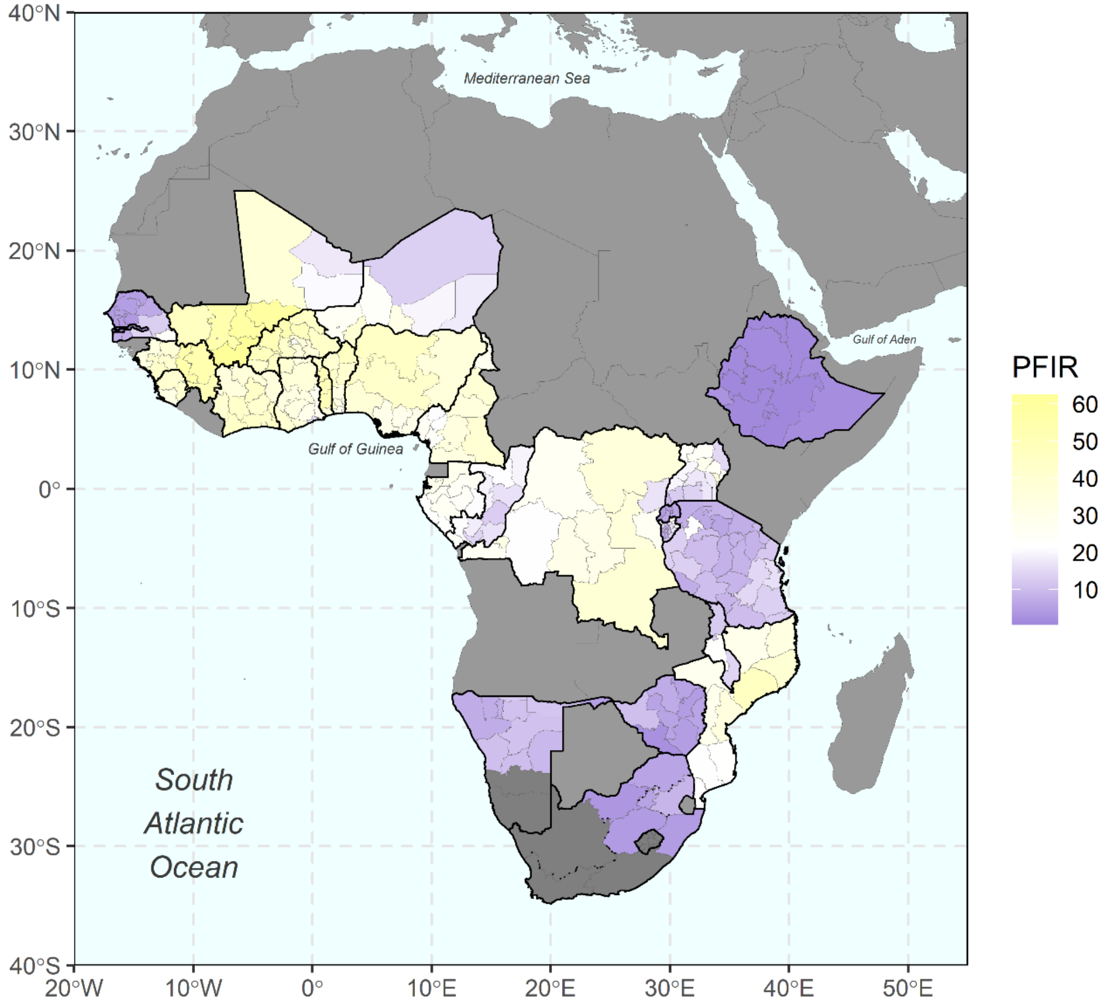

Figure S6. HIV mean prevalence, mean=4·7 (range is 0·2-26·3). Maps were generated using R programming environment, version 3.6.3 (https://cran.r-project.org/bin/windows/base/old/3.6.3/)

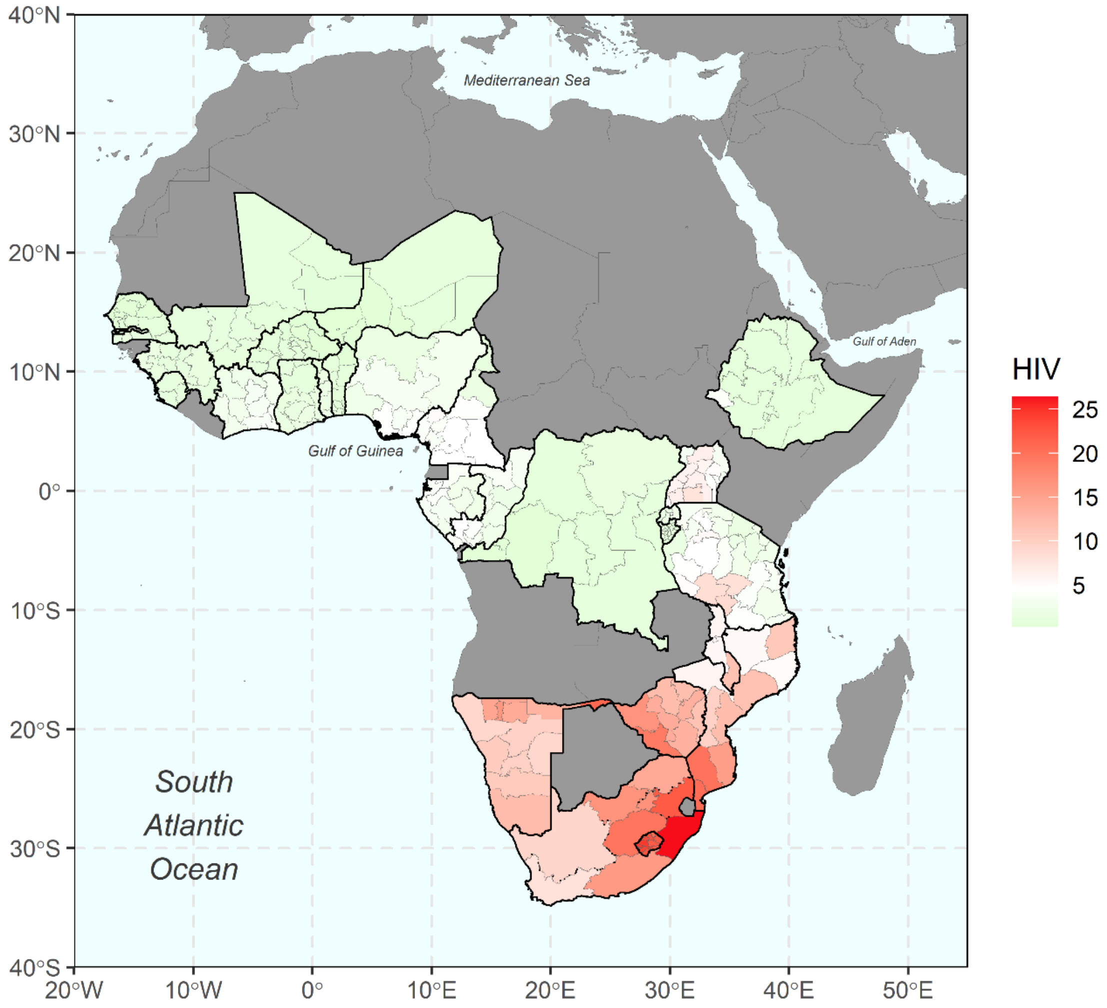

APPENDIX D: STROBE checklist

This study follows the guidelines of the Strengthening the Reporting of Observational Studies in Epidemiology [42]

STROBE item	Item No	Recommendation	Location in manuscript where items are reported
Title and abstract	1	(a) Indicate the study's design with a commonly used term in the title or the abstract	(a) Both in title and abstract (methods and findings section)
		(b) Provide in the abstract an informative and balanced summary of what was done and what was found	(b) This was done
**Introduction**			
Background/rationale	2	Explain the scientific background and rationale for the investigation being reported	Introduction, paragraph 1, 2
Objectives	3	State specific objectives, including any prespecified hypotheses	Introduction, paragraph 3
**Methods**			
Study design	4	Present key elements of study design early in the paper	Methods, paragraph 1-3
Setting	5	Describe the setting, locations, and relevant dates, including periods of recruitment, exposure, follow-up, and data collection	Methods, paragraph 1

Participants	6	(a) Give the eligibility criteria, and the sources and methods of selection of participants	Methods, paragraph 2
Variables	7	Clearly define all outcomes, exposures, predictors, potential confounders, and effect modifiers. Give diagnostic criteria, if applicable	Methods, paragraph 3
Data sources/ measurement	8*	For each variable of interest, give sources of data and details of methods of assessment (measurement). Describe comparability of assessment methods if there is more than one group	Methods, paragraph 3
Bias	9	Describe any efforts to address potential sources of bias	
Study size	10	Explain how the study size was arrived at	Appendix A
Quantitative variables	11	Explain how quantitative variables were handled in the analyses. If applicable, describe which groupings were chosen and why	Methods, paragraph 2. Appendix A
Statistical methods	12	(a) Describe all statistical methods, including those used to control for confounding	Methods, paragraph 4, 5
		(b) Describe any methods used to examine subgroups and interactions	
		(c) Explain how missing data were addressed	
		(d) If applicable, describe analytical methods taking account of sampling strategy	Methods, paragraph 2
		(e) Describe any sensitivity analyses	
**Results**			

Participants	13*	(a) Report numbers of individuals at each stage of study—eg numbers potentially eligible, examined for eligibility, confirmed eligible, included in the study, completing follow-up, and analysed	Results, paragraph 1
		(b) Give reasons for non-participation at each stage	
		(c) Consider use of a flow diagram	Appendix A
Descriptive data	14*	(a) Give characteristics of study participants (eg demographic, clinical, social) and information on exposures and potential confounders	Results, paragraph 1
		(b) Indicate number of participants with missing data for each variable of interest	Results, table 1
Outcome data	15*	Report numbers of outcome events or summary measures	Results, table 1
Main results	16	(a) Give unadjusted estimates and, if applicable, confounder-adjusted estimates and their precision (eg, 95% confidence interval). Make clear which confounders were adjusted for and why they were included	Results, paragraph 2. Appendix B
		(b) Report category boundaries when continuous variables were categorized	NA
		(c) If relevant, consider translating estimates of relative risk into absolute risk for a meaningful time period	NA

Other analyses	17	Report other analyses done—eg analyses of subgroups and interactions, and sensitivity analyses	Results, paragraph 3
**Discussion**			
Key results	18	Summarise key results with reference to study objectives	Discussion, paragraph 1
Limitations	19	Discuss limitations of the study, taking into account sources of potential bias or imprecision. Discuss both direction and magnitude of any potential bias	Discussion, paragraph 5
Interpretation	20	Give a cautious overall interpretation of results considering objectives, limitations, multiplicity of analyses, results from similar studies, and other relevant evidence	Discussion, paragraph 1-4
Generalisability	21	Discuss the generalisability (external validity) of the study results	Discussion, paragraph 6
**Other information**			
Funding	22	Give the source of funding and the role of the funders for the present study and, if applicable, for the original study on which the present article is based	Mentioned in the acknowledgements section.

REFERENCES

1 ICF International. Demographic and Health Survey Sampling and Household Listing Manual. (ICF International,, Calverton, Maryland, USA, 2012).
2 Ministère à la Présidence chargé de la Bonne Gouvernance et du Plan - MPBGP, Ministère de la Santé Publique et de la Lutte contre le Sida - MSPLS, Institut de Statistiques et d'Études Économiques du Burundi - ISTEEBU & ICF. Burundi Troisième Enquête Démographique et de Santé 2016-2017. (MPBGP, MSPLS, ISTEEBU, and ICF, Bujumbura, Burundi, 2017).
3 Institut National de la Statistique et de l'Analyse Économique & ICF. République Du Bénin Ciquième Enquête Démographique et de Santé au Bénin (EDSB-V) 2017-2018. (INSAE/Benin and ICF, Cotonou, Bénin, 2019).
4 Institut National de la Statistique et de la Démographie - INSD/Burkina Faso & ICF International. Burkina Faso Enquête Démographique et de Santé et à Indicateurs Multiples (EDSBF-MICS IV) 2010. (Institut National de la Statistique et de la Démographie - INSD/Burkina Faso and ICF International, Calverton, Maryland, USA, 2012).
5 Institut National de la Statistique - INS/Côte d' Ivoire & ICF International. Côte d' Ivoire Enquête Démographique et de Santé et à Indicateurs Multiples 2011-2012. (INS/Côte d' Ivoire and ICF International, Calverton, Maryland, USA, 2013).
6 Institut National de la Statistique/INS & ICF. République du Cameroun Enquête Démographique et de Santé 2018. (INS and ICF, Yaoundé, Cameroun, 2020).
7 Centre National de la Statistique et des Études Économiques - CNSEE/Congo & ICF International. Congo Enquête Démographique et de Santé 2011-2012. (CNSEE and ICF International, Calverton, Maryland, USA, 2012).
8 Ministère du Plan et Suivi de la Mise en œuvre de la Révolution de la Modernité - MPSMRM/Congo, Ministère de la Santé Publique - MSP/Congo & ICF International. République Démocratique du Congo Enquête Démographique et de Santé (EDS-RDC) 2013-2014. (MPSMRM, MSP, and ICF International, Rockville, Maryland, USA, 2014).
9 Central Statistical Agency - CSA/Ethiopia & ICF. Ethiopia Demographic and Health Survey 2016. (CSA and ICF, Addis Ababa, Ethiopia, 2017).
10 Direction Générale de la Statistique - DGS/Gabon & ICF International. Gabon Enquête Démographique et de Santé 2012. (Direction Générale de la Statistique - DGS/Gabon and ICF International, Calverton, Maryland, USA, 2013).
11 The Gambia Bureau of Statistics - GBOS & ICF International. The Gambia Demographic and Health Survey 2013. (GBOS and ICF International, Banjul, The Gambia, 2014).
12 Ghana Statistical Service - GSS, Ghana Health Service - GHS & ICF International. Ghana Demographic and Health Survey 2014. (GSS, GHS, and ICF International, Rockville, Maryland, USA, 2015).
13 Institut National de la Statistique & ICF. Guinea Demographic and Health Survey (EDS V) 2016-18. (INS/Guinea and ICF, Conakry, Guinea, 2019).
14 Ministry of Health/Lesotho & ICF International. Lesotho Demographic and Health Survey 2014. (Ministry of Health/Lesotho and ICF International, Maseru, Lesotho, 2016).

15 Institut National de la Statistique - INSTAT, Cellule de Planification et de Statistique Secteur Santé-Développement & ICF. Mali Demographic and Health Survey 2018. (INSTAT/CPS/SS-DS-PF and ICF, Bamako, Mali, 2019).
16 Ministerio da Saude - MISAU/Moçambique, Instituto Nacional de Estatística - INE/Moçambique & ICF International. Moçambique Inquérito Demográfico e de Saúde 2011. (MISA/Moçambique, INE/Moçambique and ICF International, Calverton, Maryland, USA, 2013).
17 National Statistical Office/Malawi & ICF. Malawi Demographic and Health Survey 2015-16. (National Statistical Office and ICF, Zomba, Malawi, 2017).
18 Ministry of Health and Social Services - MoHSS/Namibia & ICF International. Namibia Demographic and Health Survey 2013. (MoHSS/Namibia and ICF International, Windhoek, Namibia, 2014).
19 Institut National de la Statistique - INS/Niger & ICF International. Niger Enquête Démographique et de Santé et à Indicateurs Multiples (EDSN-MICS IV) 2012. (INS/Niger and ICF International, Calverton, Maryland, USA, 2013).
20 National Population Commission - NPC & ICF. Nigeria Demographic and Health Survey 2018 - Final Report. (NPC and ICF, Abuja, Nigeria, 2019).
21 National Institute of Statistics of Rwanda, Ministry of Finance and Economic Planning/Rwanda, Ministry of Health/Rwanda & ICF International. Rwanda Demographic and Health Survey 2014-15. (National Institute of Statistics of Rwanda, Ministry of Finance and Economic Planning/Rwanda, Ministry of Health/Rwanda, and ICF International, Kigali, Rwanda, 2016).
22 Agence Nationale de la Statistique et de la Démographie - ANSD/Sénégal & ICF. Senegal: Enquête Démoggraphique et de Santé Continue (EDS-Continue) 2017. (ANSD and ICF, Dakar, Sénégal, 2018).
23 Statistics Sierra Leone - SSL & ICF International. Sierra Leone Demographic and Health Survey 2013. (Freetown, Sierra Leone, SSL and ICF International, 2014).
24 Ministry of Health, C. D., Gender, Elderly and Children - MoHCDGEC/Tanzania Mainland,, Ministry of Health - MoH/Zanzibar, National Bureau of Statistics - NBS/Tanzania, Office of Chief Government Statistician - OCGS/Zanzibar & ICF. Tanzania Demographic and Health Survey and Malaria Indicator Survey 2015-2016. (MoHCDGEC, MoH, NBS, OCGS, and ICF, Dar es Salaam, Tanzania, 2016).
25 Ministère de la Planification, d. D. e. d. l. A. d. T.-M. T., Ministère de la Santé - MS/Togo & ICF International. Togo Enquête Démographique et de Santé 2013-2014. (MPDAT/Togo, MS/Togo and ICF International, Rockville,Maryland, USA, 2015).
26 Uganda Bureau of Statistics - UBOS & ICF. Uganda Demographic and Health Survey 2016. (UBOS and ICF, Kampala, Uganda, 2018).
27 National Department of Health & ICF. South Africa Demographic and Health Survey 2016. (National Department of Health - NDoH - ICF, Pretoria, 2019).
28 Zimbabwe National Statistics Agency & ICF International. Zimbabwe Demographic and Health Survey 2015: Final Report. (Zimbabwe National Statistics Agency (ZIMSTAT) and ICF International, Rockville, Maryland, USA, 2016).
29 ICF. Demographic and Health Surveys Standard Recode Manual for DHS7. (ICF, Rockville, Maryland, U.S.A., 2018).
30 (WHO), W. H. O. Guidelines for Drinking-water Quality. 631 (World Health Organization (WHO), 2017).

31 Bhatt, S. *et al.* The effect of malaria control on Plasmodium falciparum in Africa between 2000 and 2015. *Nature* **526**, 207-211, doi:10.1038/nature15535 (2015).
32 (IHME), I. f. H. M. a. E. (Institute for Health Metrics and Evaluation (IHME), Seattle, United States, 2019).
33 Abioye, A. I., Andersen, C. T., Sudfeld, C. R. & Fawzi, W. W. Anemia, Iron Status, and HIV: A Systematic Review of the Evidence. *Advances in Nutrition* **11**, 1334-1363, doi:10.1093/advances/nmaa037 (2020).
34 Weiss, D. J. *et al.* A global map of travel time to cities to assess inequalities in accessibility in 2015. *Nature* **553**, 333-336, doi:10.1038/nature25181 (2018).
35 Zuur, A. I., Elena N. Walker, Neil Saveliev, Anatoly A. Smith, Graham M. *Mixed Effects Models and Extensions in Ecology with R*. 1 edn, 574 (Springer-Verlag New York, 2009).
36 Blangiardo, M., Cameletti, M., Baio, G. & Rue, H. Spatial and spatio-temporal models with R-INLA. *Spatial and Spatio-temporal Epidemiology* **4**, 33-49, doi:https://doi.org/10.1016/j.sste.2012.12.001 (2013).
37 R: A Language and Environment for Statistical Computing v. 3.5.2 (R Foundation for Statistical Computing, Vienna, Austria, 2018).
38 survey: analysis of complex survey samples v. R package version 3.35-1 (2019).
39 Lu, Z. Handbook of spatial statistics Alan E. Gelfand, Peter J. Diggle, Montserrat Fuentes and Peter Guttorp (eds), Chapman & Hall/CRC, Boca Raton, 2010. No. of pages: xii+607. ISBN: 978-1-4200-7287-7. *Statistics in Medicine* **30**, 899-900, doi:10.1002/sim.4159 (2011).
40 Lindgren, F. & Rue, H. Bayesian Spatial Modelling with R-INLA. *2015* **63**, 25, doi:10.18637/jss.v063.i19 (2015).
41 SpatialEpi: Methods and Data for Spatial Epidemiology v. R package version 1.2.3 (2018).
42 von Elm, E. *et al.* The Strengthening the Reporting of Observational Studies in Epidemiology (STROBE) Statement: Guidelines for Reporting Observational Studies. *Epidemiology* **18**, 800-804 (2007).
